# Large-scale analysis of transcriptional *cis*-regulatory modules reveals both common features and distinct subclasses

**DOI:** 10.1186/gb-2007-8-6-r101

**Published:** 2007-06-05

**Authors:** Long Li, Qianqian Zhu, Xin He, Saurabh Sinha, Marc S Halfon

**Affiliations:** 1 Department of Biochemistry, State University of New York at Buffalo, Buffalo, NY 14214, USA; 2 Department of Biological Sciences, State University of New York at Buffalo, Buffalo, NY 14214, USA; 3 Department of Computer Science, University of Illinois Urbana-Champaign, Urbana, IL 61801, USA; 4 New York State Center of Excellence in Bioinformatics and the Life Sciences, Buffalo, NY 14203, USA; 5 Department of Molecular and Cellular Biology, Roswell Park Cancer Institute, Buffalo, NY 14263, USA

## Abstract

**Background:**

Transcriptional *cis*-regulatory modules (for example, enhancers) play a critical role in regulating gene expression. While many individual regulatory elements have been characterized, they have never been analyzed as a class.

**Results:**

We have performed the first such large-scale study of *cis*-regulatory modules in order to determine whether they have common properties that might aid in their identification and contribute to our understanding of the mechanisms by which they function. A total of 280 individual, experimentally verified *cis*-regulatory modules from *Drosophila *were analyzed for a range of sequence-level and functional properties. We report here that regulatory modules do indeed share common properties, among them an elevated GC content, an increased level of interspecific sequence conservation, and a tendency to be transcribed into RNA. However, we find that dense clustering of transcription factor binding sites, especially homotypic clustering, which is commonly believed to be a general characteristic of regulatory modules, is rather a feature that belongs chiefly to a specific subclass. This has important implications for current computational approaches, many of which are biased toward this subset. We explore two new strategies to assess binding site clustering and gauge their performances with respect to their ability to detect all 280 modules and various functionally coherent subsets.

**Conclusion:**

Our findings demonstrate that *cis*-regulatory modules share common features that help to define them as a class and that may lead to new insights into mechanisms of gene regulation. However, these properties alone may not be sufficient to reliably distinguish regulatory from non-regulatory sequences. We also demonstrate that there are distinct subclasses of *cis*-regulatory modules that are more amenable to *in silico *detection than others and that these differences must be taken into account when attempting genome-wide regulatory element discovery.

## Abstract 2

Analysis of 280 experimentally-verified *cis*-regulatory modules from *Drosophila *reveal features both common to all and unique to distinct subclasses of modules.

## Background

Regulated spatial and temporal control of gene expression is a fundamental process for all metazoans, and much of this regulation occurs through the interaction of transcription factors (TFs) with specific *cis*-regulatory DNA sequences. The best-defined of these regulatory elements are promoters, which are easily identified based on their position surrounding the transcription start sites (TSSs) of their associated genes [1]. However, promoters comprise just a small fraction of important functional *cis*-regulatory sequences. A large amount of gene regulation is mediated by *cis*-regulatory elements that are distal to the promoter and organized in a modular fashion (reviewed by [2]). Each module regulates a particular temporal-spatial pattern of gene expression that is a subpart of the entire expression pattern of its associated gene; at the molecular level, each contains a series of binding sites for a specific complement of TFs. Often referred to as 'enhancers', these elements can lie hundreds of kilobases away from the promoter and can be located 5', 3', or within the intron of their own or a non-associated gene. Here, we use the more generic term '*cis*-regulatory module' (CRM) to refer both to enhancers and to other classes of regulatory sequences.

The number of CRMs in the genome is believed to be very high; Davidson [2] suggests that there might be five-to-ten times as many individual CRMs in the genome as there are genes. It has become increasingly apparent that polymorphisms and mutations in CRMs play a major role as producers of normal phenotypic variation, as inducers of birth defects and chronic diseases, and as a powerful evolutionary driving force [2-4]. Despite their prevalence and importance, however, much less is known about CRMs in general than about promoters. This is largely due to the difficulties involved in identifying CRMs, which until recently has been possible only through a dedicated empirical approach of testing sequence fragments for regulatory activity in a reporter gene assay, either in transgenic animals or an appropriate cell culture system. In the past several years, a number of computational approaches for CRM identification have been attempted, with varying degrees of success (for example, [5-22]). Broadly speaking, most of these methods fall into either or both of two classes: those based on sequence alignment, or those dependent on transcription factor binding site (TFBS) clustering. In the first, putative CRMs are predicted based on conservation of non-coding sequences between two or more related species. In the latter, CRMs are defined as regions containing a particular number and/or combination of specific TFBSs. Considerations regarding these approaches and their variations have been reviewed elsewhere [23-28] and will not be discussed at length here. However, it is important to note that all of these methods have at their core an underlying assumption that CRMs contain common properties that will facilitate their discovery, that is, interspecific conservation or TFBS clustering.

From numerous examples, we know that both of these assumptions at times hold true. Many known CRMs are well-conserved in related species [22,29,30], and most of the extensively studied CRMs, in particular the enhancers of the *Drosophila *early patterning genes, consist of a dense cluster of TFBSs containing multiple occurrences of TFBSs for a small number of transcription factors [31-33]. This latter property is sometimes referred to as 'homotypic clustering' of TFBSs due to the repeated numbers of similar sites [34]. Nevertheless, there are also characterized CRMs that do not contain one or the other, or even both, of these properties. Late pair-rule expression of the *Drosophila runt *gene, for instance, is regulated by a diffuse CRM spread over 5 kb of sequence that is poorly conserved in distantly related *Drosophila *species [35,36]. Although this is typically viewed to be the exception rather than the rule, evidence to support this belief is thin and suffers from significant ascertainment bias: since many known CRMs were discovered based on one of these two properties, there is naturally an overrepresentation of conserved CRMs with clustered TFBSs. Thus, the actual extent to which these are common or unusual CRM characteristics remains undetermined.

We recently constructed a database of *cis*-regulatory elements in *Drosophila melanogaster*, the REDfly database, which contains records for over 650 experimentally verified positive-acting CRMs drawn from the published literature [37]. These CRMs are responsible for regulating the expression of a diverse set of genes in many different tissues and stages of development. Here, we present the results of our first large-scale analysis of the REDfly CRMs to define properties that are common to CRMs as a class, and those that are present only in specific CRM subsets. In the first section of the paper we describe the general sequence properties of *Drosophila *CRMs and show that CRMs are more GC-rich and evolutionarily conserved compared to other non-coding sequences, and are likely to be transcribed into RNA. Our data indicate that while CRMs have these distinct common properties as a class, they are difficult to distinguish from non-CRMs as individual sequences. In the second part of the paper we focus on TFBS clustering and show that homotypic TFBS clustering is prevalent only in certain CRM groups. We also undertake two new approaches to CRM discovery, neither of which are biased by any prior knowledge of binding sites, and show that these too favor the subclasses of CRMs with the greatest amount of TFBS clustering. Throughout, we consider the impact of the unknown fraction of CRMs present in unannotated non-coding sequence on all aspects of CRM discovery and analysis.

## Results

### Basic characteristics of the REDfly CRMs

#### Number and size

At the time we initiated this study, the REDfly database [37] contained 544 records of known *Drosophila *CRMs. We chose for analysis the subset of these that were non-overlapping and that were less than 2,100 base-pairs (bp) in length. This length cutoff captured 75% of the non-overlapping CRMs and was imposed based on our concern that CRMs of greater than 2 kb of sequence or so would contain large amounts of non-functional sequence (that is, that a more minimal CRM would exist within the larger sequence that had not yet been experimentally isolated). There were 280 CRMs associated with 148 genes, with an average length of 760 bp (Figure S1-1A in Additional data file 1), that met these criteria and are referred to hereafter as the 'REDfly analysis CRMs'. A detailed listing of these CRMs can be found in Additional data file 2. Analysis of a subset of these CRMs, in which only those ≤1,000 bp in length were used, gave essentially identical results to those reported below (data not shown).

#### Functional roles

In order to determine the breadth of the functional spectrum covered by the genes associated with the REDfly analysis CRMs, we looked at the Gene Ontology (GO) terms for these genes and at the stages and tissues in which the REDfly analysis CRMs regulate gene expression. GO term designations to which ≥10% of the CRM-associated genes map are shown in Table S1-1 in Additional data file 1. Although there is a bias toward CRMs associated with genes encoding transcription factors (>50%) and for genes involved in development (>80%), embryonic, larval, and adult stages of development are all represented (Figure S1-1B in Additional data file 1). A large variety of tissues are also represented (Figure S1-1C in Additional data file 1). Of these, embryonic blastoderm is the most heavily covered tissue (19%), followed by neuronal tissue (13%). An alternative breakdown of tissue representations is provided in Figure S1-2 in Additional data file 1.

#### Genomic location

Figure S1-1D in Additional data file 1 describes the location of the REDfly analysis CRMs with respect to the TSS of their associated genes: 61% of the CRMs are located 5' to the annotated TSS; 13% of the CRMs overlap the promoter or are completely contained within the first 500 bp 5' of the TSS while 38% begin more than 500 bp 5'. 13% of the CRMs are downstream of the annotated 3' end of their genes, while 16% lie within introns. The vast majority of these are within the first (50%) or second (27%) introns, but CRMs are found within sixth and seventh introns as well (Figure S1-3 in Additional data file 1).

Genes with multiple transcripts present a particular problem for assigning the location of CRMs; when the transcripts are generated from alternative promoters, a CRM can be upstream of one TSS, but in an intron of another. As a result, 10% of the REDfly analysis CRMs have a 'mixed' upstream and intronic location. It is generally unknown whether the CRMs influence the expression of all or only a subset of the transcripts with which they are associated.

### CRMs have an elevated GC content

**Figure 1:**
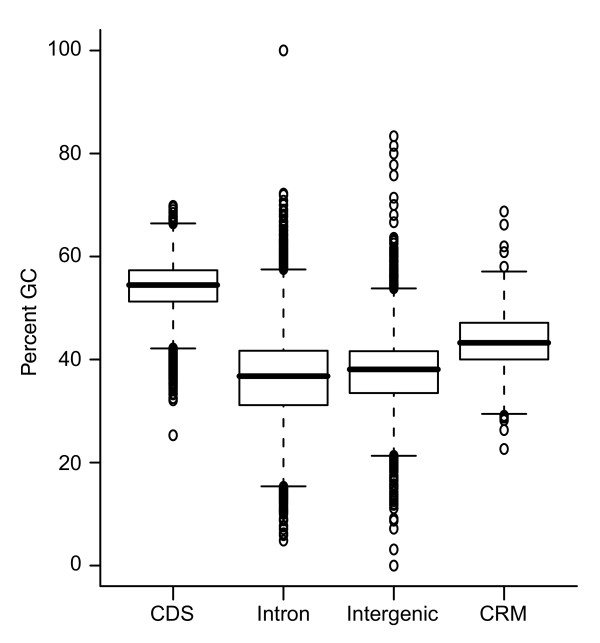
GC content of the REDfly analysis CRMs as well as coding, intronic, and intergenic sequences.

**Figure 2:**
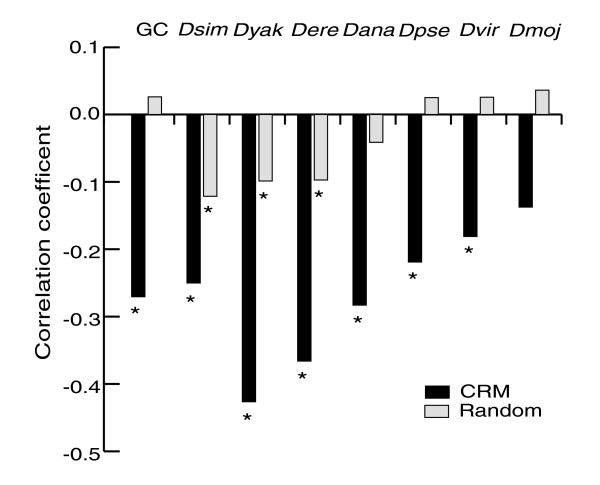
Correlations between CRM length and GC content (column 1) and degree of sequence conservation with seven *Drosophila *species. Values given are the Spearman correlation coefficients. Black bars indicate CRM sequences, gray bars indicate size-matched randomly drawn non-coding sequence. Asterisks signify that the correlation is statistically significant (Bonferroni-adjusted *P *< 0.05). *Dsim*, *D. simulans*; *Dyak*, *D. yakuba*; *Dere*, *D. erecta*; *Dana*, *D. ananassae*; *Dpse*, *D. pseudoobscura*; *Dvir*, *D. virilis*; *Dmoj*, *D. mojavensis*.

We measured the average GC content of the REDfly analysis CRMs and compared it to that of coding sequences, intergenic regions, and introns (Figure 1). It has previously been shown that the GC content in coding sequences is higher than that of non-coding sequences [38,39], and that *Drosophila *promoters tend to be AT-rich [40]. Surprisingly, we found that the REDfly analysis CRMs have a higher average GC content than other intergenic or intronic sequence, although a lower GC content than coding regions (mean 0.45 (standard deviation (SD) 0.06) versus 0.37 (0.07), rank sum test *P *< 1e-16; 0.45 (0.06) versus 0.54 (0.05), rank sum test *P *< 1e-16). This does not appear to be the result of a higher density of TF binding sites present in the CRMs, as an analysis of the footprinted binding sites contained in the FlyReg database [41] shows that they have an average GC content similar to that in non-CRM intergenic sequence (data not shown). No differences in the results were observed when various tissue- or stage-specific subsets were used in place of the entire 280 REDfly analysis CRMs (data not shown). A moderate negative correlation exists between CRM length and GC content (Figure 2; Spearman's ρ = -0.27, *P *< 9e-06). Size-matched random non-coding sequences are uncorrelated with GC content (Figure 2b; Spearman's ρ = 0.03, *P *= 0.28). Assuming that longer introns are likely to contain more CRMs than short introns [42], the higher GC content of CRMs versus non-regulatory non-coding sequence may help to account for the observations by Haddrill *et al*. [43], who saw both a positive correlation between intron length and GC content, and a negative correlation between GC content and sequence divergence between *D. melanogaster *and *D. simulans *introns (as CRMs are more highly conserved; see below).

### CRMs are more highly conserved than non-regulatory sequences

**Figure 3:**
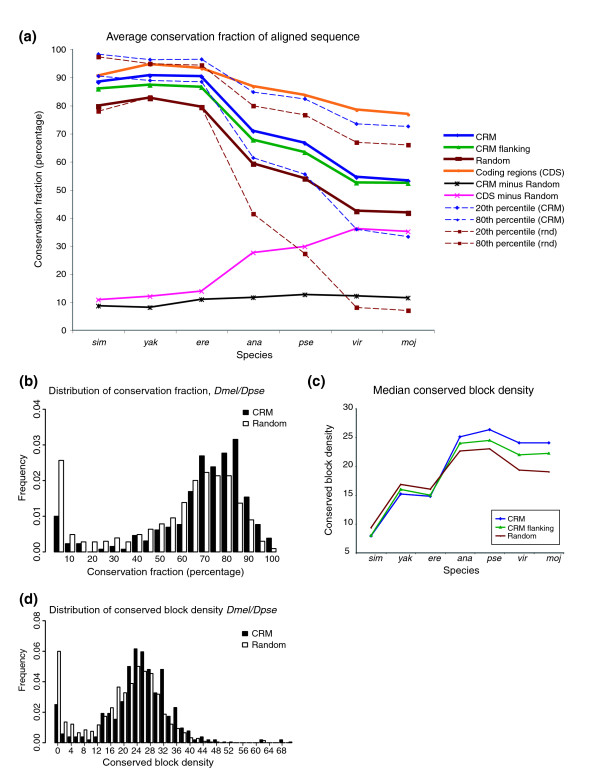
Sequence conservation properties of the REDfly analysis CRMs. **(a) **Average fraction of aligned bases between *D. melanogaster *and each of the other species for the CRMs (blue), CRM flanking sequences (green; ± 1 kb to each side of the CRM; see text), coding regions (orange; based on 2,000 genes; see Materials and methods), and size-matched randomly selected non-coding sequences (red). Dashed lines indicate the 20% and 80% percentile values for the CRMs and random sequences. Also indicated are the 'differences' in conservation between CRMs and random non-coding sequences (black) and between coding sequences and random non-coding sequences (pink). Species abbreviations are as given in the legend to Figure 3. A similar graph showing the fraction of aligned 'identical' bases is given in Figure S3-1 in Additional data file 3. **(b) **Histogram of the conservation fraction for CRMs (black bars) and random non-coding sequences (white bars) for *D. melanogaster *aligned with *D. pseudoobscura*. Histograms for the other species are shown in Figure S3-2 in Additional data file 3. **(c) **Median conserved block density for each of the species aligned to *D. melanogaster*. Blocks are defined as ungapped regions of seven or more nucleotides with ≥75% identity. Shown are block densities for CRMs (blue), CRM flanking regions (green), and size-matched randomly selected non-coding sequences (red). **(d) **Histogram of the distribution of conserved block density for CRMs (black bars) and random non-coding sequences (white bars) for *D. melanogaster *aligned with *D. pseudoobscura*. Histograms for the other species are shown in Figure S3-3 in Additional data file 3.

Functional sequences are expected to be conserved among related species, a property that has been used successfully for the identification of CRMs in many organisms (reviewed by [44]). This approach has worked particularly well in vertebrates, for which a wide range of related species have been sequenced. However, while it is clear that conserved sequences frequently contain CRMs, it is less clear how often CRMs lie in non-conserved sequences, nor how many conserved sequence regions do not contain CRMs. To begin to address these questions, we constructed pairwise alignments between the REDfly CRM sequences in *D. melanogaster *and *D. simulans*, *D. yakuba*, *D. erecta*, *D. ananassae*, *D. pseudoobscura*, *D. mojavensis*, and *D. virilis *(more closely to more distantly related, respectively; [45]) using DIALIGN [46]. DIALIGN was chosen due to its strong performance in a previous assessment of alignment of simulated non-coding sequences [47]. We assessed both the conservation of the CRM sequences themselves and the conservation of sequences up to 1 kb to each side of the CRM and compared these alignments with alignments of size-matched, randomly selected non-coding sequences. We assessed conservation in terms of both fraction of aligned bases and degree of nucleotide identity between two sequences; both measures gave similar results (Figure 3; Figure S3-1 in Additional data file 3; data not shown).

We find that CRMs are on average significantly more well-conserved than randomly chosen non-coding sequences (Figure 3a; Figure S3-1 in Additional data file 3; Kolmogorov-Smirnov test, Bonferroni-corrected *P *< 7e-07). The sequences flanking the CRMs are generally less conserved than the CRMs but more conserved than the random sequences. Some of the increased conservation of the flanking sequences relative to randomly drawn ones may be due to the presence of coding regions within these sequences. However, this is unlikely to account for the entire observed difference as the majority of the CRMs are sufficiently far from their associated coding regions that the flanking sequences contain only non-coding DNA (data not shown). We speculate that most of the difference is due either to a greater likelihood for the adjacent sequences to contain additional (as yet unidentified) CRMs, or to the gradual loss of regulatory function in these sequences due to binding site turnover (for example, [48-50]). Interestingly, we find that although as expected, the degree of CRM conservation decreases with increased evolutionary distance, the difference between the amount of conservation in CRMs versus random sequences remains essentially constant (Figure 3a). This is in marked contrast to the difference between coding and random sequences, which increases steadily with evolutionary distance. The different behaviors of the two types of functional sequences appear to be due to a faster rate of divergence in CRMs versus coding sequences. As with GC content, no differences in the results for any of the conservation-related properties were observed when various tissue- or stage-specific subsets were used in place of the entire set of 280 REDfly analysis CRMs (data not shown).

Despite the clear difference in mean conservation fraction between CRMs and random non-coding sequence, the distributions of the two sets are highly overlapping (Figure 3b; Figure S3-2 in Additional data file 3). Therefore, degree of sequence conservation would appear to be an ineffective way of reliably distinguishing regulatory from non-regulatory sequences. We note, however, that an unknown fraction of the random non-coding sequence we use will actually contain regulatory elements and might in addition contain other currently unannotated functional sequences such as missed first exons and micro-RNAs. The higher this fraction, the more likely we are to be underestimating the true amount of separation between the regulatory and non-regulatory sequences. We return to this point in more detail in the Discussion.

As we observed for GC content, CRM length and conservation fraction are negatively correlated, with more closely related species generally having a greater degree of correlation than more distantly related ones (Figure 2; *P *< 0.05). We also observe a weak but statistically significant negative correlation for randomly selected non-coding sequences in the most closely related species. This is in contrast to results recently reported by Halligan and Keightley [51], who found that non-coding sequence length is negatively correlated with divergence. The difference may be due to the different scale of the two analyses: our study is mainly looking at much shorter sequences.

Although the magnitude of the difference in sequence conservation between CRMs and random non-coding sequences is relatively constant among all the analyzed species, the *pattern *of conservation differs. We looked at conserved sequence blocks of 7 bp or more with ≥75% identity in CRMs, their flanking sequences, and random non-coding sequences. While the length of conserved blocks does not vary significantly among these groups (with the exception of *D. simulans*; Figure S3-3 in Additional data file 3; data not shown), there is a significant difference in the *density *of conserved blocks in the more diverged species. In these species, CRMs have more blocks per kilobase than do random non-coding sequences (Figure 3c; Kolmogorov-Smirnov test, Bonferroni-corrected *P *< 0.003). As we saw for overall conservation, sequences adjacent to the CRMs fall in between the CRMs and the random sequences. Again, however, the distributions are highly overlapping, suggesting that conserved block density also is not a reliable discriminator between regulatory and non-regulatory sequences (Figure 3d; Figure S3-4 in Additional data file 3). Our results differ slightly from those of Papatsenko *et al*. [52], who observed an increased number of long (>20 bp) conserved blocks in CRM sequences when comparing *D. melanogaster *and *D. pseudoobscura*. The differences are likely due to the fact that that study defined blocks as having 100% identity versus our looser standard of 75% identity. Nevertheless, our overall conclusions are in agreement with those of Papatsenko *et al*. [52].

### Ultraconserved elements are overrepresented in CRMs

Several recent studies have remarked on the presence of 'ultraconserved' elements and other highly conserved regions in both vertebrate and invertebrate genomes [19,53,54]. Ultraconserved elements (uc-elements) are long stretches of sequence (≥50 bp) that are perfectly conserved over tens of millions of years of evolution. The majority of these are associated with genes encoding TFs and other regulators of development, and it has been hypothesized that uc-elements lying in non-coding regions might serve as all or parts of *cis*-regulatory modules [54]. Glazov *et al*. [55] have identified uc-elements conserved between *D. melanogaster *and *D. pseudoobscura*, and we examined the extent of overlap between these uc-elements and the REDfly analysis CRMs. Of the 20,301 non-coding uc-elements conserved between the two fly species, 84 overlap a REDfly analysis CRM by greater than 15 bp. On average, a mean of 98% (11% SD) of each of these 84 uc-element sequences is contained within a CRM. In all, 61 of the REDfly analysis CRMs (22%) contain at least one uc-element, with 28% of these containing two or more (Additional data file 4). This is significantly greater overlap than we find for uc-elements in size-matched random non-coding sequence controls (17% of sequence 'elements'; Fisher's exact *P *< 0.04). The overrepresentation of uc-elements within CRMs is even more apparent when the total amount of ultraconserved base-pairs is considered: 2.5% of the total REDfly analysis CRM sequence is ultraconserved, versus only 1.8% of size-matched random non-coding sequence (Fisher's exact *P *< 2.2e-16). Again, we note that these data are likely to understate the differences in the regulatory and non-regulatory populations due to the presence of an unknown number of regulatory and/or coding elements in the randomly selected sequence.

### CRM sequences are transcribed with high frequency

Recent transcriptional profiling studies using whole-genome tiled microarrays in a number of organisms have revealed that a much larger fraction of the genome than previously appreciated is transcribed into RNA [56-62] (reviewed by [63]). We used the microarray data of Manak *et al*. [64], which covers the *Drosophila *genome at 35 bp resolution, to determine whether or not the REDfly analysis CRMs are transcribed. We found that over 35% (99/280) of the CRMs were transcribed versus only 23% (3,194/14,000) of size-matched randomly selected non-coding sequences (*P *< 4.05e-07 by two-sample test of proportions). Thus, CRM sequences are transcribed with higher frequency than non-CRM sequences. Data from a second *Drosophila *tiled microarray experiment [58] are consistent with this result, although differences in microarray design prevent a direct comparison of the datasets (see Additional data file 5, Table S5-1 and Figure S5-1).

### A modified Fluffy-tail test distinguishes CRM from non-CRM sequences

We next turned our attention to a property often assumed to be common to the majority of CRMs, that of TFBS clustering. Abnizova *et al*. [65] have proposed a method, the Fluffy-tail test (FTT), that relies on homotypic TFBS clustering to identify CRMs. Like a number of other CRM discovery methods (for example, [34,66,67]), the FTT uses similar nucleotide subsequences as a proxy for related binding sites. The FTT score is based on the size of the largest group of 'similar words' - related nucleotide subsequences - in a CRM sequence and was reported to have excellent performance at distinguishing CRMs from non-regulatory non-coding sequences when analyzing 60 *Drosophila *CRMs (Figure S6-1 in Additional data file 6, columns 1 and 2). We therefore decided to make use of the FTT to test the underlying assumption that dense homotypic TFBS clustering is a general feature of CRMs.

**Figure 4:**
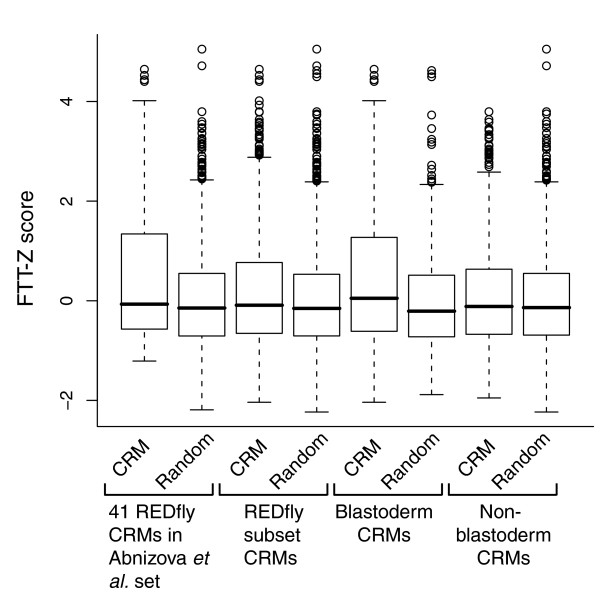
Results from the FTT-Z test. Boxplots indicate the median (heavy bar) and first and third quartiles of the data (boxed area). Details are provided in the text.

We developed a revised version of the FTT, which we refer to as the FTT-Z (see Materials and methods), that performs similarly to the original test but eliminates a problem in which the score is confounded with the length of the sequence being analyzed (Figures S6-2 and S6-1 in Additional data file 6, columns 3 and 4). There are 41 of the REDfly analysis CRMs present in the original FTT training set. When we applied the FTT-Z to these 41 CRMs, we found that the separation between the CRMs and random non-coding sequence was very poor, suggesting that the FTT-Z score does not provide a good method for distinguishing regulatory from non-regulatory sequences (Figure 4, columns 1 and 2). However, there is a significant difference in the mean scores between the two groups (CRMs, 0.55 ± 0.09 (mean ± standard error of the mean); random non-coding -0.01 ± 0.07; rank sum test *P *< 2.5e-05). We therefore went on to apply the test to all of the REDfly analysis CRMs. Once again, we found that the difference in the mean scores was statistically significant between CRMs and random non-coding sequences (0.15 ± 0.03 versus 0.02 ± 0.02; rank sum test *P *< 0.02), but the separation remained very poor (Figure 4, columns 3 and 4).

### Blastoderm CRMs are different from other CRMs

Although both sets of CRMs are significantly different from random sequence, the mean score when using all of the REDfly analysis CRMs is significantly smaller than the score using the 41 CRM training set (rank sum test *P *< 3.7e-04). We noted that close to 80% of the 41 CRMs are CRMs that regulate gene expression in the early embryonic blastoderm (referred to hereafter as 'blastoderm CRMs') and wondered whether this might account for the difference in scores. Therefore, we compared separately the 80 REDfly analysis CRMs annotated as being blastoderm CRMs and the remaining 200 non-blastoderm CRMs to both random non-coding sequence and to each other. While the blastoderm CRMs are significantly different from random sequence (Figure 4, columns 5 and 6; 0.36 ± 0.06 versus 0.01 ± 0.05; rank sum test *P *< 8.2e-05), the non-blastoderm CRMs and random sequence are indistinguishable (Figure 4, columns 7 and 8; 0.07 ± 0.03 versus 0.03 ± 0.03; rank sum test *P *< 0.14). Furthermore, the blastoderm and non-blastoderm CRMs are significantly different from one another (Figure 4, columns 5 and 7; rank sum test *P *< 4.7e-04). We therefore conclude that the differences observed between the REDfly analysis CRMs and random non-coding sequences are due mainly to the presence of the blastoderm CRMs. These data suggest that although the blastoderm CRMs have large numbers of homotypic repeats, CRMs in general are no different from non-regulatory sequences in this regard.

We also tested whether stage- or tissue-specific categories of CRMs containing ≥15 members (Figure S1-1B, C in Additional data file1) have FTT-Z scores that are different from randomly selected sequences. Other than the blastoderm CRMs, only those annotated as being associated with gene expression in the ectoderm, embryo, and adult have significant differences (Table S6-1 in Additional data file 6). However, these are not mutually exclusive classes, and the 'ectoderm' and 'embryo' CRMs overlap considerably with the blastoderm CRMs. Therefore, it is probable that the high FTT-Z scores of the blastoderm CRMs account for most of differences seen in these subsets.

### Biases in CRM type found by CRM discovery algorithms

Sets of CRMs consisting primarily of blastoderm CRMs have been used to develop a number of computational approaches to CRM discovery [5,14,65-69]. Our results from the FTT-Z demonstrate that the blastoderm CRMs differ from CRMs in general in their degree of similar nucleotide subsequences. We therefore wondered if methods that were trained and tested on a blastoderm CRM dataset were biased toward discovery of CRMs with an unusually strong homotypic repeat structure. We reasoned that if this were the case, the CRMs found by these methods would have high FTT-Z scores, whereas unbiased methods would be uncorrelated with FTT-Z scores. To test for such biases, we ranked all of the REDfly analysis CRMs by FTT-Z score and assessed the median rank (highest score = 100%) of the CRMs discovered by the various other methods (Table 1). An unbiased method should have a median rank around 50% ('expected' in Table 1), while a heavily biased method would have a median rank close to 100%. We found that the previously known CRMs used in the training sets ('known') had a median rank of 90%, confirming the heavy bias toward homotypic repeats in that set. Similarly, the CIS-ANALYST method of Berman *et al*. [6] predicted CRMs with a median rank of 92%, suggesting that while effective for finding blastoderm-like CRMs with a dense subsequence repeat structure, this type of algorithm would be likely to perform poorly at discovering the majority of the known *Drosophila *CRMs. On the other hand, the Ahab algorithm used by Schroeder *et al*. [33] found CRMs with a median FTT-Z rank of only 57% and might thus provide a CRM discovery method less geared toward the fraction of CRMs with highly repeated subsequences.

**Table 1:** Performance of CRM discovery methods with respect to FTT-Z score of confirmed CRMs

Method	Reference	Median rank*
Expected	-	50%
Known^†^	-	90%
CIS-ANALYST	[6]	92%
PFR-Searcher	[67]	73%
Fly Enhancer	[13]	65%
Ahab	[33]	57%
-	[14]	39%

*Median rank of CRMs among all 280 REDfly analysis CRMs ranked by FTT-Z score. ^†^'Known' CRMs are those used as training data by either/or CIS-ANALYST or Ahab.

### A YMF-based method can distinguish CRMs from non-regulatory sequences

**Figure 5:**
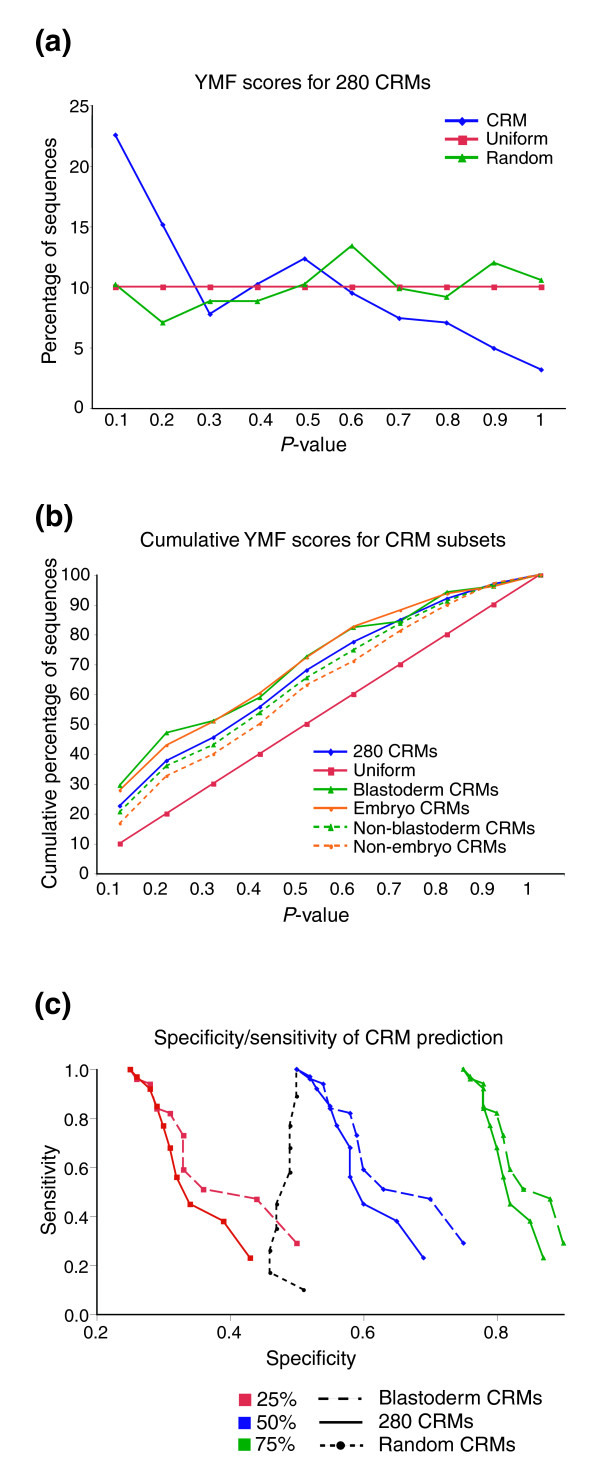
YMF scores for the REDfly analysis CRMs. **(a) **Histograms of percentage of CRMs for given *P *value ranges (YMF scores). The histogram for all 280 REDfly analysis CRMs is shown in blue ('CRMs'), for randomly selected non-coding sequences in green ('Random'), and for the random expectation ('Uniform') in red. **(b) **Cumulative histograms of YMF scores for tissue- and stage-specific CRM subsets. The entire REDfly analysis set is shown in blue and the expected uniform distribution in red. Solid green lines indicate the blastoderm CRMs, while dashed green lines represent the non-blastoderm CRMs; orange solid and dashed lines show the embryo and non-embryo CRM subsets, respectively. Note that all subsets show significant deviation from the expected uniform distribution. **(c) **Specificity/sensitivity curves for CRM prediction using YMF. Three sets of curves are shown, representing three different assumptions as to the number of CRMs present in the randomly selected background sequences: 25% CRMs (red), 50% CRMs (blue), and 75% CRMs (green). Solid lines indicate curves for the entire 280 REDfly analysis CRMs, while dashed lines show the blastoderm CRM subset. The black dashed line represents the curve for randomly selected sequences, shown for 50% background CRMs only. For each category, the random expectation is equal to the assumed number of CRMs in the background.

As an alternative approach to addressing the question of whether binding site clustering is a general property of CRMs, we ran the motif-finding program YMF [70] for each CRM. YMF identifies motifs (words representing related subsequences) that are statistically overrepresented in a sequence or set of sequences and generates a count of how many unique motifs are found. The count of overrepresented motifs for each CRM was compared to the corresponding counts from 50 size-matched randomly selected non-coding sequences, and an empirically computed *P *value was derived for each CRM (see Materials and methods). The resulting distribution of scores shows a significant bias towards low *P *values, compared to the uniform distribution of *P *values expected by chance (Figure 5a, blue versus red curves; Table 2; Kolmogorov-Smirnov test, *P *< 3.54e-11). This indicates that a CRM, on average, contains a larger number of significant motifs than a randomly chosen size-matched non-coding sequence. As a negative control, we created a collection of randomly chosen genomic sequences of the same lengths as the REDfly CRMs, and repeated the exercise. As expected, we found that the distribution of the *P *value scores is close to uniform (Figure 5a, green curve; Table 2; *P *≅ 1).

**Table 2:** Significance of YMF results for tissue/stage-specific subsets

Tissue/stage*	Number of CRMs	*P *value^†^
All REDfly analysis CRMs	280	**3.54E-11**
Random non-coding	280	1
Blastoderm	51	**6.53E-04**
Non-blastoderm	207	**1.02E-05**
Mesoderm	24	0.78
Embryo	128	**9.00E-07**
Non-embryo	123	0.07
Larva	32	1
Neuronal	22	0.31

*See Figure S1-1 in Additional data file 1). Only CRMs uniquely assigned to the tissue or stage are included here. ^†^Kolmogorov-Smirnov test. *P *values for subsets are Bonferroni-corrected. Values in bold are significant.

In light of the results from the FTT-Z indicating that the blastoderm CRMs have distinct properties, we recalculated the histogram of *P *value scores (Figure 5a) for each of several subsets of the REDfly analysis CRMs, formed on the basis of similarity of expression stages or tissue types (Table 2; Figure 5b). The blastoderm CRMs have a higher percentage of low *P *values than the CRMs in general, consistent with the idea that TFBS clustering is more prevalent in this CRM subset (*P *< 6.53e-04). Other tissue-specific subsets that were tested were not significantly different from random expectation (Table 2). One key difference from the FTT-Z results is that although the FTT-Z found that the non-blastoderm CRMs do not significantly differ from random non-coding sequences, these CRMs are still biased toward low YMF *P *values and score in a range similar to the REDfly analysis CRMs as a whole (Figure 5b; data not shown). This difference is likely the result of the different ways each method assesses TFBS clustering (see Discussion).

### Prediction of CRMs using YMF

We can use the YMF *P *value score to predict whether or not a given sequence is a CRM (see Materials and methods). Sensitivity of the prediction is based on the *P *value score used as a threshold for calling a sequence a CRM, while the specificity of prediction depends on the true proportion of CRMs in the genome. That is, we assume that some number of the random non-coding sequences are in fact currently unidentified CRMs. Under the assumption that 50% of the input sequences are CRMs, we can achieve a prediction specificity of 69% at a sensitivity of 23%, much better than the 50% specificity expected by chance. Figure 5c shows the specificity of CRM prediction expected at varying levels of sensitivity under different assumptions about genomic CRM abundance (25%, 50%, and 75% of randomly chosen genomic sequences being CRMs). Note that the blastoderm CRMs can be predicted with much better sensitivity/specificity than the other CRMs, consistent with our previous finding that they comprise a distinct CRM subclass (Figure 5c, dashed versus solid lines).

### Supervised learning and classification of CRMs versus random genomic sequences

As a third way of testing the TFBS clustering properties of CRMs, we undertook a supervised learning approach to CRM classification based on a modification of the HexDiff algorithm [66]. We used frequencies of short subsequence words to train an algorithm to discriminate CRMs from non-CRMs (see Materials and methods). The classification accuracy was evaluated in a ten-fold cross validation exercise in which the REDfly analysis CRMs were treated as the positive set and an equal number of randomly chosen genomic sequences (of the same lengths as the CRMs) used as the negative set.

A set of 175 modules (the REDfly analysis set after removing CRMs <500 bp or >2,000 bp), augmented with an equal sized 'negative' set of random sequences, could be classified correctly with an accuracy of 63.8% in a 10-fold cross-validation exercise (Table 3; Binomial test *P *< 1.9e-07). Note that this figure is not comparable to the sensitivity or specificity values given for the YMF algorithm, since an accurate prediction in this exercise requires correctly classifying both 'positive' (CRM) and 'negative' (non-CRM) samples.

**Table 3:** Results from supervised learning

Tissue/stage*	Classification accuracy	*P *value
REDfly analysis CRMs	63.8%	**1.9E-07**
Blastoderm	68.4%	**3.5E-03**
Neuronal	65.0%	0.16
Embryo	59.1%	**0.04**
Larva	42.5%	1

*See Figure S1-1 in Additional data file 1. Only CRMs uniquely assigned to the tissue or stage are included here. *P *values for subsets are Bonferroni-corrected. Values in bold are significant.

Like with the FTT and YMF methods, we also evaluated tissue- and stage-specific subsets of CRMs using this learning algorithm and a leave-one-out-cross-validation strategy. The 'blastoderm', and 'embryo' CRMs gave significantly high classification accuracy in similar cross-validation experiments (Table 3). As we saw with the other methods, the blastoderm CRMs have the most pronounced differences compared to the other CRM subsets and to the entire REDfly analysis set.

## Discussion

Two commonly held assumptions about transcriptional *cis*-regulatory modules are that their sequences are evolutionarily conserved and they contain a high degree of TFBS clustering. We present here a large-scale analysis of *Drosophila *CRMs designed to evaluate these and other CRM properties. This is the largest such study performed to-date for any metazoan; nevertheless, only about 1% of *Drosophila *genes are represented, with presumably only a subset of the CRMs for each gene. Our main conclusions can be summarized as follows: first, CRMs have distinct properties that as a group distinguish them from other types of DNA sequences, regardless of the tissues or stages in which they regulate gene expression. Second, these differences are typically not great enough to reliably classify a given unknown sequence as CRM or non-CRM. Third, TFBS clustering, and homotypic TFBS clustering in particular, can begin to provide more reliable classification of sequences as CRM or not CRM. Fourth, homotypic clustering is not a general characteristic of CRMs but rather is prevalent only in certain CRM subclasses.

### Sequence conservation

Many CRMs, particularly in vertebrates, have been discovered by virtue of sequence conservation, leaving open the possibility that the strong conservation of CRMs noted in these species may be at least partially due to ascertainment bias. As the majority of the REDfly analysis CRMs were discovered by means other than an assessment of conservation (data not shown), they present a useful test set for evaluating this bias. Our results agree with studies of much smaller sets of *Drosophila *CRMs [6,71]. Similar to those, we see a statistically significant increase in the fraction of conserved sequence in CRMs versus non-CRMs, but with a distribution not too different from that of randomly selected sequences. One caveat lies in the fact that the REDfly CRMs are heavily biased toward those associated with genes with important functions in development, as there is evidence from studies in vertebrates that these CRMs are more likely to be conserved than others [29]. Overall levels of conservation of CRM sequences might thus be lower than what we have observed here.

The difference in degree of conservation between coding and non-coding sequences increases with evolutionary distance. Surprisingly, this is not the case for CRMs and their flanking sequences, both of which retain a roughly constant degree of difference in conservation fraction compared to random non-coding sequences. Thus, CRM sequences diverge more rapidly than coding sequences, but in proportion with the overall degree of sequence divergence of non-coding DNA. This may be due to a general conflation of CRMs and what we call random non-coding sequence: our CRMs might contain large amounts of non-regulatory non-coding sequence, or the randomly selected non-coding sequences might contain a large fraction of CRM sequence. We favor the view that both of these phenomena are occurring.

Support for the idea that the REDfly CRMs contain a substantial amount of non-regulatory sequence is provided by the negative correlations that we observe between CRM length and both GC content and sequence conservation. That is, longer CRMs are more like random non-coding sequences in their sequence properties than are shorter CRMs. We interpret this to mean that many of the REDfly CRMs are 'too long' - they have not been defined down to minimal functional sequences. However, we cannot rule out the (non-exclusive) possibilities that all of the CRM DNA is functional but either contains redundant elements that are more free to mutate, or constrained at a non-sequence level (for example, spacing between TFBSs).

### What fraction of non-coding sequence consists of CRMs?

There is also good evidence to suggest that a significant fraction of the *Drosophila *non-coding DNA is functional and may harbor large numbers of CRMs. Halligan and Keightley [51] have recently estimated that greater than 50% of non-coding sequence is subject to selective constraint and, therefore, presumably functional, while Nelson *et al*. [72] have shown that genes with complex expression patterns are associated with longer flanking non-coding sequences than genes with simple expression patterns. Moreover, the *Drosophila *genome has a high rate of DNA loss in unconstrained sequences through deletion events [73]. Taken together, these data argue that the *Drosophila *genome is compact and contains a high proportion of regulatory sequence.

Both non-functional sequence included in our CRM set and, more importantly, a high density of CRMs within non-coding sequences, have important implications for the results we have presented here, as either feature will lead to underestimation of the observed sequence properties. That is, the more that our CRMs are contaminated with non-CRM sequence, and vice versa, the less good will be the separation that we detect between the two sequence classes. Therefore, although our results suggest that CRMs and non-regulatory non-coding sequences are not clearly distinguishable, an improved knowledge of the background fraction of CRMs in non-coding sequence would potentially reveal a greater separation. Unfortunately, until a truly unbiased empirical assessment of regulatory activity over an extensive selection of non-coding DNA is conducted, there may not be sufficient data to make a proper estimate of the true CRM fraction.

### Transcription of CRM sequences

Whole-genome tiling microarray experiments and detailed EST sequencing projects have repeatedly revealed that much higher percentages of the genomes of multiple organisms are transcribed than originally believed [56-62], although the functional significance of this transcription remains unclear [63] and even controversial [74]. Our analysis suggests that a substantial number of intergenic and intronic transcribed sequences could be CRMs. Transcription of regulatory sequences has been observed previously, notably in the *Drosophila bithorax *complex (BX-C) and in the locus control regions (LCRs) of several vertebrate gene clusters [75-80]. Johnson *et al*. [81] suggest that transcription at the *β-globin *LCR is a consequence of RNA polymerase II recruitment by the LCR, but other studies suggest that active transcription of the CRM is required for gene activation (for example, [82]). In the BX-C, CRM transcription is restricted to the tissues in which the respective CRMs are active [75], but it is unknown whether or not most CRM transcription is temporally and spatially regulated or if it correlates with activity or inactivity of the CRM. Further study of transcribed CRM sequences at much higher spatial and temporal resolution than has currently been conducted will be necessary before these and related questions can be answered.

### TFBS clustering

A commonly held assumption about CRMs is that they contain tightly clustered binding sites for one or several TFs, with most of the sites represented multiple times (that is, homotypically clustered) [2,31]. As there also exist examples of other types of CRM organization, an important question becomes determining which is the exception, and which the rule [83]. Since the REDfly CRMs span a broad range of regulatory systems, with most of the relevant transcription factors not characterized, we faced the technical challenge of assessing TFBS clustering without knowing the actual binding sites. We therefore used several different methods to count occurrences of similar words (motifs) as a surrogate for measuring the extent of TFBS clustering. The FTT looks only at the subsequence with the highest incidence in the CRM and thus provides a measure of homotypic TFBS clustering [65]. YMF, on the other hand, considers how many different motifs are overrepresented in the CRM [70]. YMF is, therefore, simultaneously a measure of homotypic clustering (each TFBS identified must be present at greater than background levels) and heterotypic clustering (multiple significant TFBSs must be present). Both methods clearly separate the blastoderm CRMs from the non-blastoderm CRMs, suggesting that not only do blastoderm CRMs tend to have more homotypic TFBS repeats than other CRMs (FTT results) but also that they frequently contain a larger number of distinct binding sites (YMF results). However, while non-blastoderm CRMs are indistinguishable from random non-coding sequences by the FTT, YMF clearly differentiates between the two. Heterotypic TFBS clustering may thus be a more common property of CRMs than extensive homotypic clustering, which appears to be a property mainly of specific CRM subclasses.

### Biological significance of homotypic clustering

The prevalence of homotypic TFBS clustering in the CRMs responsible for regulating transcription in the early embryonic blastoderm may relate directly to the biology of early fly development. The use of CRMs consisting of multiple binding sites with varying affinities has long been recognized as an important component of the mechanism by which genes can determine their position with respect to a morphogen gradient [84,85]. This is precisely the situation found in the early fly embryo, which develops as a syncytium in which patterns of gene expression are largely determined by TF concentration gradients (reviewed by [86]).

Consistent with the idea that homotypic clustering is associated with interpreting positional information based on morphogen gradients, we note that a number of non-blastoderm REDfly CRMs associated with morphogen-responsive genes have high FTT-Z scores indicative of homotypic TFBS clustering. For instance, CRMs for the *Ance/race *gene, which responds to morphogen gradient signaling during embryogenesis [87], and for the *salm *and *bi/omb *genes, which respond to morphogen gradient signaling in the wing imaginal disc [88], rank in the top half of the FTT-Z scores (percent ranks = 75.2%, 69.1%, and 54.1%, respectively). Nevertheless, not all genes in these classes have high levels of TFBS clustering, suggesting that other means are also used to ensure correct readout of morphogen concentrations [89].

In circumstances where gene expression is not regulated through morphogen gradients, the need for dense TFBS clusters may be less important, consistent with our finding that homotypic clustering is not a general CRM property. In a cellular environment, TFs are unable to diffuse from one spatial gene expression domain to another as they do in the syncytial blastoderm, and sharp boundaries of TF activation domains can be maintained by other methods. The apparently more widespread presence of heterotypic TFBS clusters fits with the idea that transcriptional regulation is highly combinatorial, with CRMs acting to integrate the input from multiple signaling pathways and tissue-specific selector proteins [3,90].

### Implications for CRM discovery methods

Our large-scale study of CRM sequences provides a starting point for re-evaluating existing methods for computational CRM discovery and designing new approaches. Most methods that have been developed for computational discovery of CRMs are based on the assumption that CRMs have common properties that can provide a signal for their identification, primarily either sequence conservation or TFBS clustering (see reviews in [23-28]). We have demonstrated here that CRMs do indeed share common properties, but the separation between the CRM and non-CRM populations is too poor to allow for successful discrimination. While this separation might be improved in organisms with a less highly compact genome than *Drosophila*, tests of conserved sequences in vertebrates also suggest that there, too, conservation alone is not a sufficient marker of regulatory sequence (for example, [91]). Nevertheless, despite not being enough to discriminate between CRMs and non-regulatory sequences on their own, these features can contribute to an overall scoring function for CRM identification.

Importantly, our results demonstrate that distinct subsets of CRMs can have specific properties not shared by all regulatory modules but which can be highly effective for CRM discovery. Therefore, one-size-fits-all approaches to CRM discovery are likely to be less effective than methods tailored toward specific subsets. A favorable strategy might therefore be to use methods that make use of a set of coexpressed genes for training data, such as the PFR-Sampler/Searcher programs [67], consistent with our finding that a supervised learning approach provided greater sensitivity than the other methods. Focusing on only a subset of CRMs might also make it easier to incorporate the detection or use of 'grammatical' rules, such as constraints in the spacing of TFBS pairs, into CRM discovery algorithms [9,11,92,93]. Our study also highlights the value of having a large and diverse set of known CRMs to use for training and evaluation purposes, something that for the higher eukaryotes currently exists only for *Drosophila*. Generating similar collections for human and other model organisms should remain an important future goal.

## Materials and methods

### Gene Ontology term mapping

GO terms for the CRM-associated genes were determined using GOTermMapper [94].

### Sequences

For comparisons of sequence conservation between CRMs and random non-coding sequences, five non-coding sequences of the same length as the CRM were randomly chosen from the genome of *D. melanogaster *for each CRM; values are the average value of the five sequences. Because Halligan and Keightley [51] have reported a correlation between the length of intergenic and intron sequences and their degree of conservation, it is possible that artifacts could be introduced if a CRM and its size-matched non-coding sequences were drawn from regions of different lengths. We tested this possibility for a small set of CRMs (*n *= 30, lengths <1 kb different were considered identical) and found that there was no difference in the results if the random sequences were drawn from a similarly sized non-coding region or not (*P *values for the various species ranged from 0.24-0.94 by paired *t*-test). Therefore, random sequences were chosen without regard to intergenic or intron sequence length. Conservation values for coding sequences are based on a random selection of 2,000 non-overlapping coding regions. Assignment of sequence as coding or non-coding was based on release 4.2 of the *Drosophila *genome annotation.

### Alignments

Globally aligned sequence regions for each of the species used were obtained from the multiple alignments available from the Berkeley Comparative Genomics project [95] using the version 2 alignment of builds *DroMel_4*, *DroYak_1*, *DroAna_20041206*, *DroEre_20041028*, *DroMoj_20041206*, *DroPse_1*, *DroSim_20040829*, *and DroVir_20041029*. Sequence data for *D. simulans *and *D. yakuba *were produced by the Genome Sequencing Center at Washington University School of Medicine in St Louis [96], those for *D. ananassae*, *D. erecta*, *D. mojavensis *and *D. virilis *by Agencourt Bioscience [97], and those for *D. pseudoobscura *by the Human Genome Sequencing Center at Baylor College of Medicine [98]. 'Flanking' sequences were defined by extending each CRM (or size-matched random sequence) 1 kb on each end, or until the sequence could no longer be aligned, whichever was shorter. Each of these sequence regions was then aligned pairwise with the *D. melanogaster *sequence using DIALIGN [46] with the following parameters: -n -it -fa. Four of the REDfly analysis CRMs could not be cleanly assigned to orthologous sequence regions and were omitted from the assessments of conserved sequence (*Dfd_EAE-C*, *gt_-1_construct*, *siz_loner/CG32434-PE*, *tld_promoterfusion*).

### Transcribed CRMs

Significance of CRM transcription was assessed using 50 size-matched sets of randomly selected non-coding sequence and a two-sample *z*-test of proportions such that:

$$z=\frac{p_{1}-p_{2}}{\sqrt{\left(\frac{1}{n_{1}}+\frac{1}{n_{2}}\right)\left(p\right)\left(1-p\right)}}$$

where:

$$p=\frac{p_{1}n_{1}+p_{2}n_{2}}{n_{1}+n_{2}}$$

where *p*_1 _= proportion of transcribed CRMs, *p*_2 _= proportion of transcribed random sequences, and *n*_1 _and *n*_2 _are the total number of sequences for CRMs and random, respectively.

### FTT

To reduce running time, the FTT MATLAB program [65] was rewritten in C++. We created two versions of the FTT, one in which the GC content for the randomized ('shuffled') sequences was adjusted probabilistically (that is, each base chosen according to the probability of it being a specific nucleotide based on the nucleotide distribution of the original sequence), identical to the FTT of Abnizova et al. [65], and one in which GC content was held fixed by randomly rearranging the actual bases of the original sequences. Both of these versions performed identically and both gave similar results to the MATLAB version (data not shown).

The FTT-Z test was modified from the FTT as follows: for each CRM, the F score from the FTT was calculated as in the original program, except that we increased the number of shuffled sequences ('*r*') from *r *= 50 to *r *= 1000. To calculate the FTT-Z score, we then selected 500 randomly drawn non-coding sequences of the same length and from the same chromosome as the CRM (except in the case of CRMs on chromosome 4, for which random sequence was drawn from chromosome 2) and calculated the F score for each of these. A Z-score was then calculated as:

$$\frac{F_{C}-{\bar{F}}_{R}}{\sigma_{R}}$$

where *F*_*C *_is the F score of the CRM sequence and ${\bar{F}}_{R}$ and *σ*_*R *_are the mean and standard deviation of the F scores of the random sequences, respectively. Source code for the FTT-Z is available upon request.

For both the FTT and the FTT-Z, we conducted the tests a total of six times using either the CRMs or six independently generated sets of size-matched random non-coding sequences. The data reported in the text and in Figures 1 and 2 represent all six repetitions of the test.

### Computation of YMF score

YMF [70] was run on every window of length 1,000 (with shifts of 100) in the given sequence, with motif length 6 and up to 1 degenerate symbol allowed. The count of motifs with a reported z-score ≥3 is the 'YMF count score'. Random non-coding sequences of the same length were chosen from the D. *melanogaster *genome (Release 3.1), the YMF count score was computed for each of these, and an empirical *P *value computed as the fraction of random sequences that scored greater than or equal to the given sequence's score. This empirical *P *value is called the 'YMF score'. All sequences, including the randomly chosen ones, were tandem repeat masked with the 'Tandem Repeats Finder' program [99] with parameters '2 3 5 80 10 25 500' before processing.

### Estimation of sensitivity and specificity of CRM prediction

A sequence is classified as a CRM if its YMF score (empirical *P *value) is below a threshold τ. Given a set of true CRMs, the sensitivity is simply the fraction of these classified as CRMs. The specificity of prediction is calculated by making assumptions about the fraction of randomly selected non-coding sequences that are actually CRMs, as follows. Let S be an input sequence, let T_S _be the event that S is classified as being a CRM (because its YMF score was below threshold), let G_S _be the event that S is a true CRM, and let B_S _be the event that it is not a true CRM. The specificity is defined as the probability that a sequence S classified as a CRM is indeed a true CRM. That is, the specificity is given by:

$$\mathrm{Pr}(G_{S}|T_{S})=\frac{\mathrm{Pr}(T_{S}|G_{S})\mathrm{Pr}(G_{S})}{\mathrm{Pr}(T_{S}|G_{S})\mathrm{Pr}(G_{S})+\mathrm{Pr}(T_{S}|B_{S})\mathrm{Pr}(B_{S})}$$

Note that Pr(*T*_*S*_|*B*_*S*_) = τ, since the probability that a random (non-CRM) sequence has an empirical *P *value (YMF score) below threshold τ is itself τ. Pr(*T*_*S*_|*G*_*S*_) is computed from the true (known) CRMs as the fraction of them that were classified as CRMs. Finally, we can make varying assumptions about Pr(*G*_*S*_), the prior probability that a sequence S is a CRM, and set Pr(*B*_*S*_) = 1 - Pr(*G*_*S*_) to obtain the specificity at threshold τ. We show results under three different assumptions about the true proportion of CRMs: Pr(*G*_*S*_) = 0.25, 0.5, 0.75.

### Classification of CRM versus random genomic sequences using supervised learning

We implemented a variation of the HexDiff algorithm [66] to classify CRM sequences. The training data for our classifier consists of a set of CRMs ('positive' sequences) and a set of equally many random genomic fragments ('negative' sequences) with lengths matching the CRM lengths. (Sequences were not repeat masked for this exercise, and sequences with lengths <500 bp or >2,000 bp were excluded from this analysis.) We first find a set of discriminative words (hexamers) from the training data; the discriminative power *s*(*w*) of a word *w*, is measured by:

$$\ln\frac{f_{p}(w)}{f_{b}(w)}$$

where *f*_*p*_(*w*) is the frequency of *w *in the CRM sequences and *f*_*b*_(*w*) is the frequency of *w *in the background, taken to be the entire release 3 *Drosophila *genomic sequence. After ranking all the words by this score, we choose a set *W *to be the top κ words, where κ is determined from the training data (as described below). To score a sequence, we scan this sequence with a sliding window of size 500 and set the score of this sequence to be the maximum score of all windows. The score of a window is defined as:

∑*n*(*w*) *s*(*w*)

for all *w *in the set *W*, where *n*(*w*) is the number of occurrences of *w *in this window and *s*(*w*) is the discriminative score we computed in the training stage. A sequence with score above some threshold τ is predicted to be a CRM. The values of κ and τ are determined as those which, if used, lead to maximum prediction accuracy (least number of misclassified sequences) on the training sequences. In our experiments, we tried all values of κ from 200 to 400, with a step size of 50. Finally, each sequence in the test data is predicted as a CRM or not, using the values of κ and τ learned from training data.

## Additional data files

The following additional data are available with the online version of this paper. Additional data file 1 contains data on sequence-level properties of the REDfly analysis CRMs, including Figures S1-1 (basic properties of the CRMs), S1-2 (alternative mapping to tissues), S1-3 (distribution of intronic CRMs), and Table S1-1 (GO terms). Additional data file 2 is a GFFv3 file giving the locations and additional information on the REDfly analysis CRMs. Additional data file 3 contains Figures S3-1 through S3-4 with additional data on the evolutionary conservation of the REDfly CRMs. Additional data file 4 is a table showing the overlap of REDfly CRMs with ultra-conserved sequences. Additional file 5 provides data on transcription of CRM sequences based on the microarray data of Stolc *et al*. [58] and includes Table S5-1 and Figure S5-1. Additional data file 6 illustrates differences between the original FTT and the revised FTT-Z (Figures S6-1, S6-2) and contains Table S6-1, which lists CRM subsets with significant FTT-Z scores.

## Supplementary Material

Additional data file 1 Figures S1-1 (basic properties of the CRMs), S1-2 (alternative mapping to tissues), S1-3 (distribution of intronic CRMs), and Table S1-1 (GO terms). Click here for file

Additional data file 2 GFFv3 file giving the locations and additional information on the REDfly analysis CRMs. Click here for file

Additional data file 3 Figures S3-1 through S3-4 and additional data. Click here for file

Additional data file 4 Overlap of REDfly CRMs with ultra-conserved sequences. Click here for file

Additional data file 5 Table S5-1 and Figure S5-1. Click here for file

Additional data file 6 Differences between the original FTT and the revised FTT-Z (Figures S6-1 and S6-2) and CRM subsets with significant FTT-Z scores (Table S6-1). Click here for file
