# Supplementary material for: Large-scale analysis of transcriptional cis-regulatory modules reveals both common features and distinct subclasses
Source: Genome Biol. 2007 Jun 5;8(6):R101. doi: 10.1186/gb-2007-8-6-r101 (PMC2394749; doi:10.1186/gb-2007-8-6-r101)
Supplement: Additional data file 1 — Figures S1-1 (basic properties of the CRMs), S1-2 (alternative mapping to tissues), S1-3 (distribution of intronic CRMs), and Table S1-1 (GO terms). [file gb-2007-8-6-r101-S1.pdf]

Additional Data File 1: Sequence-level properties of the REDfly analysis CRMs

Figure S1-1. Basic properties of the REDfly analysis CRMs. (A) Histogram of the CRM lengths. (B) Stages at which the CRMs positively regulate gene expression. CRMs can act at more than one stage. (C) Tissues in which CRMs positively regulate gene expression. CRMs can be active in more than one tissue. An alternative breakdown of tissues is given in Fig. S1-2. (D) Location of the CRMs with respect to the transcription start sites of their associated genes. Locations were determined based on *Drosophila* release 4.3 sequence annotations. For purposes of this study, we defined any CRM wholly contained within the 500 bp 5' to the TSS, or overlapping the TSS and extending not more than 500 bp 5', as "promoter proximal" and any CRM entirely located more than 500 bp 5' of the TSS as "distal." Those CRMs whose 3' end was <500 bp from the TSS but whose 5' was >500 bp from the TSS were considered "indeterminate." CRMs associated with genes with multiple transcripts were labeled "mixed" if they did not fall into the same location category for all transcripts.

Figure S1-1
Li et al.

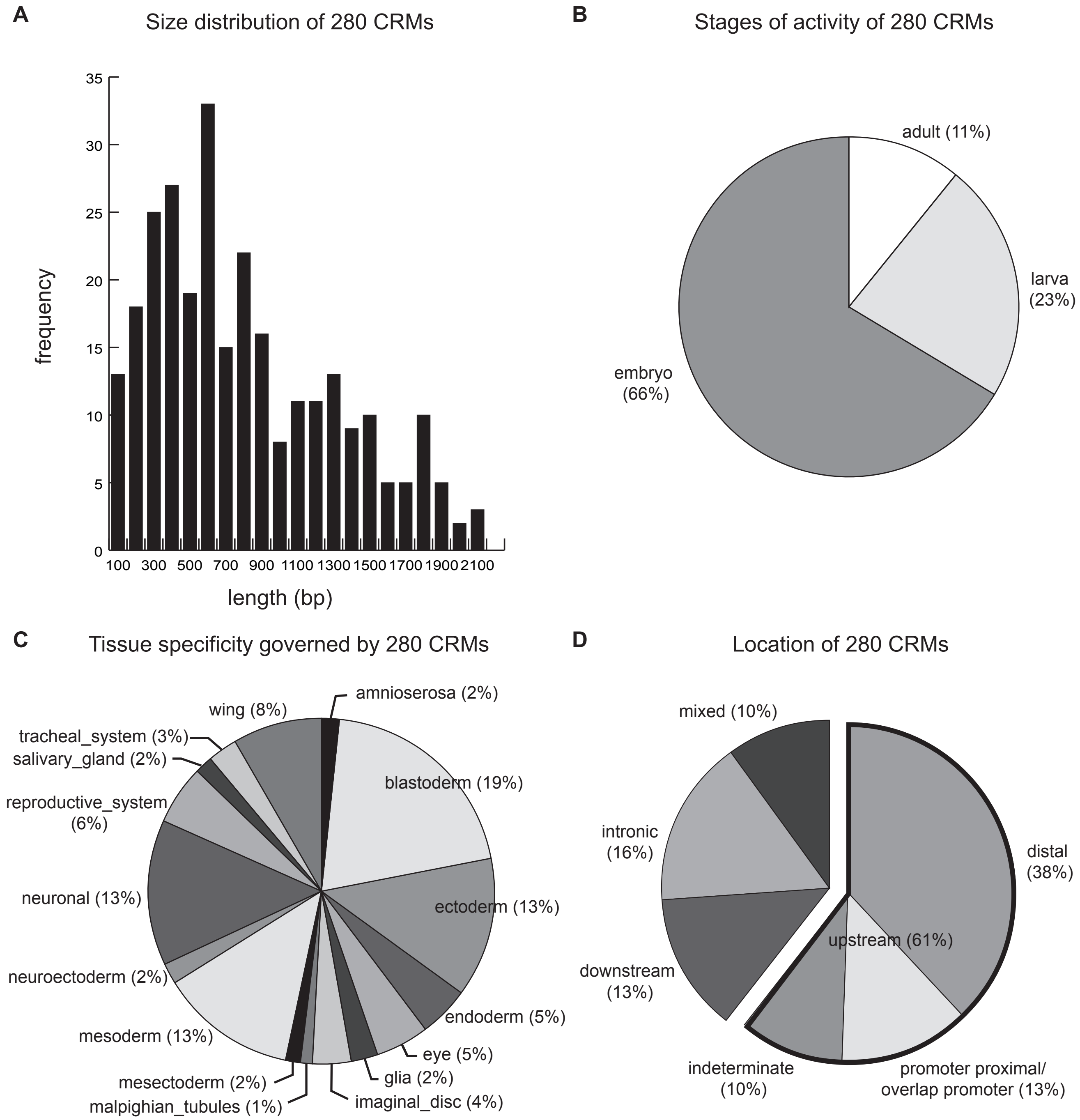

Table S1-1: GO terms representing ≥ 10% of genes associated with the REDfly analysis CRMs

GO Term	GO ID	% of Genes
**molecular function**		
binding	GO:0005488	81%
nucleic acid binding	GO:0003676	56%
transcription regulator activity	GO:0030528	54%
DNA binding	GO:0003677	49%
transcription factor activity	GO:0003700	41%
protein binding	GO:0005515	26%
signal transducer activity	GO:0004871	19%
catalytic activity	GO:0003824	19%
hydrolase activity	GO:0016787	10 %
structural molecule activity	GO:0005198	10%
**biological process**		
physiological process	GO:0007582	94%
development	GO:0007275	83%
metabolism	GO:0008152	76%
primary metabolism	GO:0044238	74%
regulation of biological process	GO:0050789	65%
nucleobase, nucleoside, nucleotide and nucleic acid metabolism	GO:0006139	60%
transcription	GO:0006350	58%
morphogenesis	GO:0009653	51%
cell differentiation	GO:0030154	46%
embryonic development	GO:0009790	44%
cell communication	GO:0007154	29%
signal transduction	GO:0007165	26%
reproduction	GO:0000003	25%
cell organization and biogenesis	GO:0016043	24%
cell proliferation	GO:0008283	19%
protein metabolism	GO:0019538	14%
behavior	GO:0007610	11%
response to biotic stimulus	GO:0009607	10%

Figure S1-2. Alternative mapping of CRMs to tissues in which they regulated gene expression (cf. Fig. S1-1C). CRMs can be active in more than one tissue.

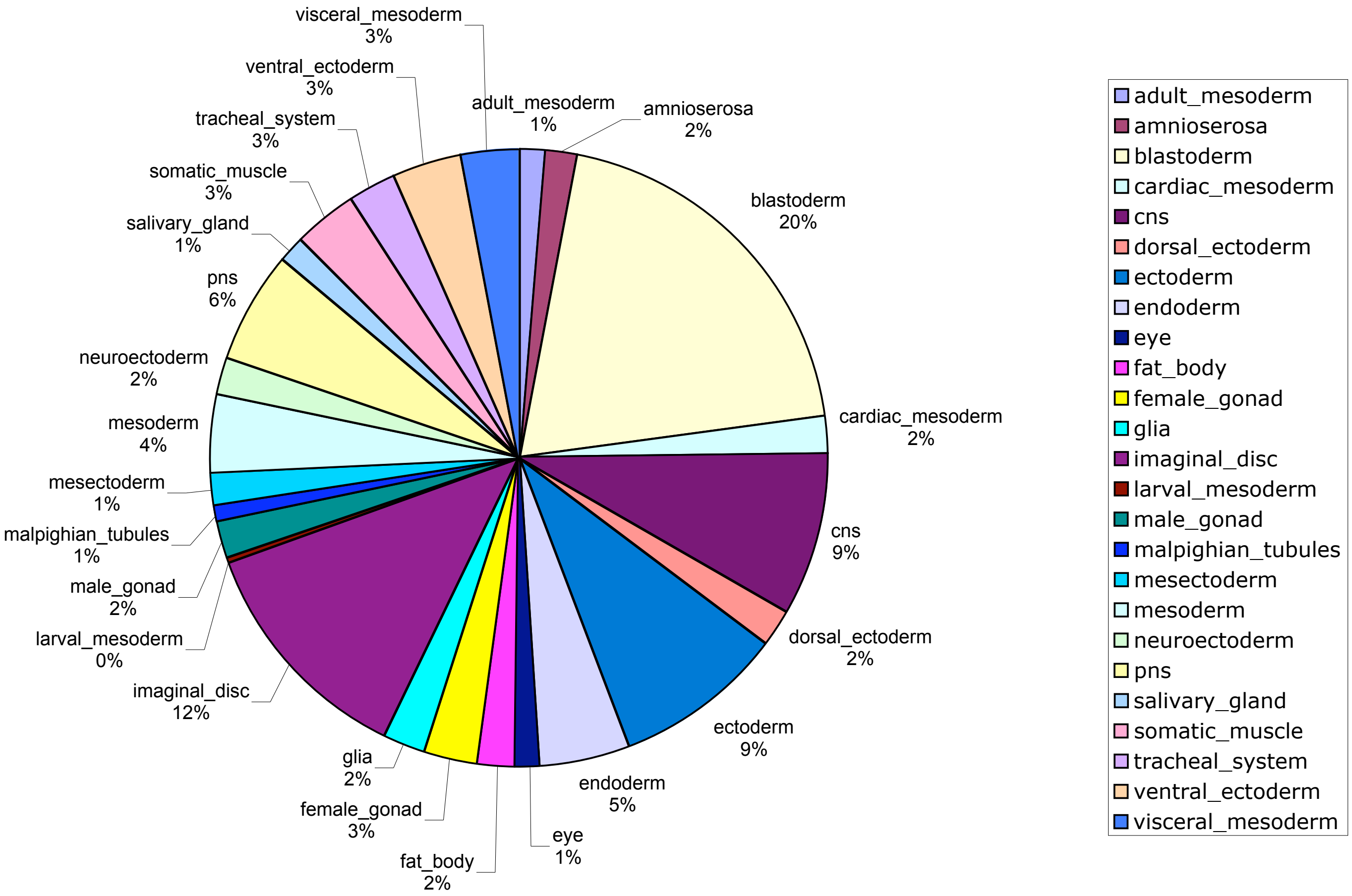

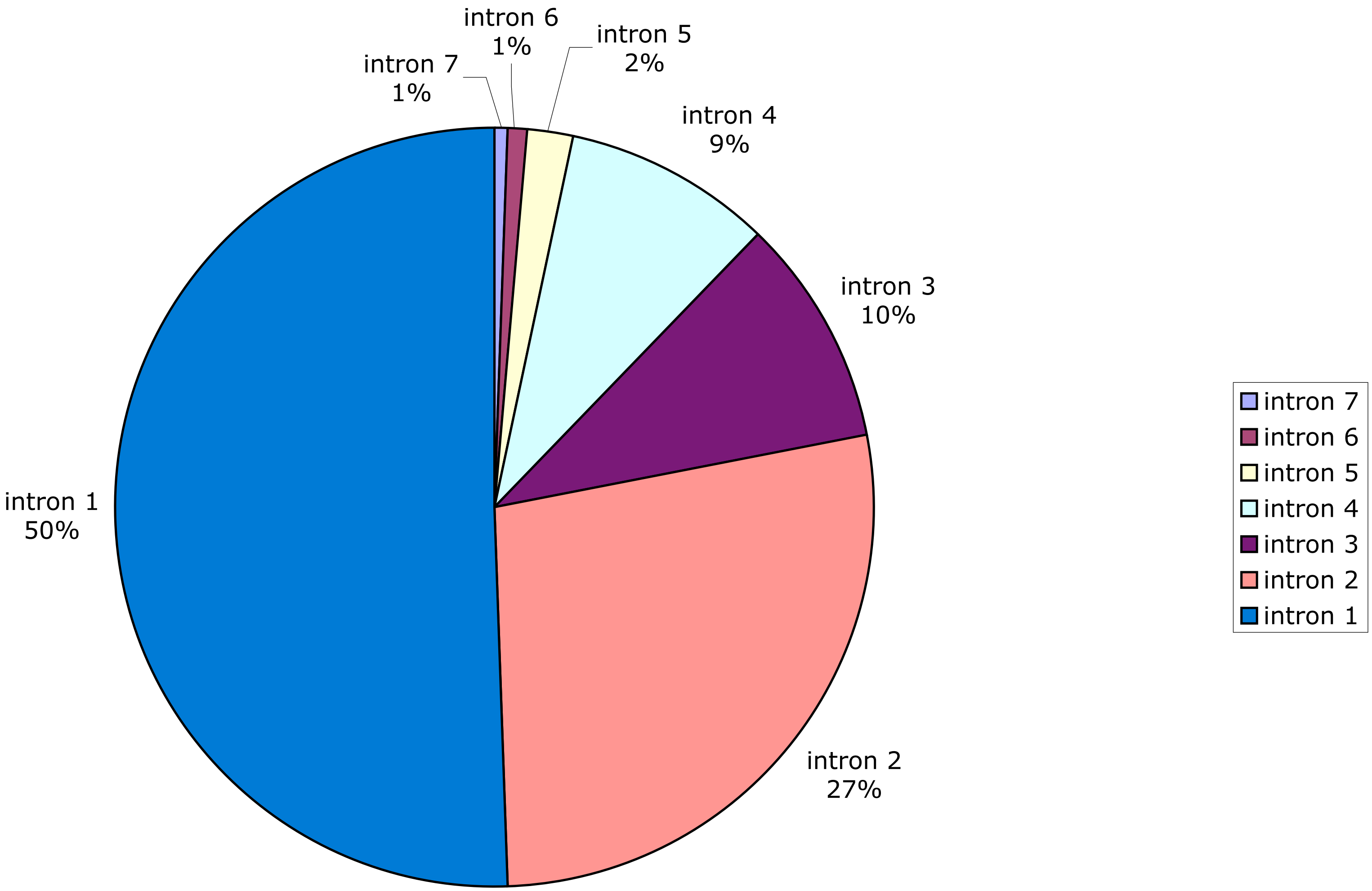

Figure S3: Distribution of REDfly subset CRMs found in introns
