# Supplementary material for: Large-scale analysis of transcriptional cis-regulatory modules reveals both common features and distinct subclasses
Source: Genome Biol. 2007 Jun 5;8(6):R101. doi: 10.1186/gb-2007-8-6-r101 (PMC2394749; doi:10.1186/gb-2007-8-6-r101)
Supplement: Additional data file 3 — Figures S3-1 through S3-4 and additional data. [file gb-2007-8-6-r101-S3.pdf]

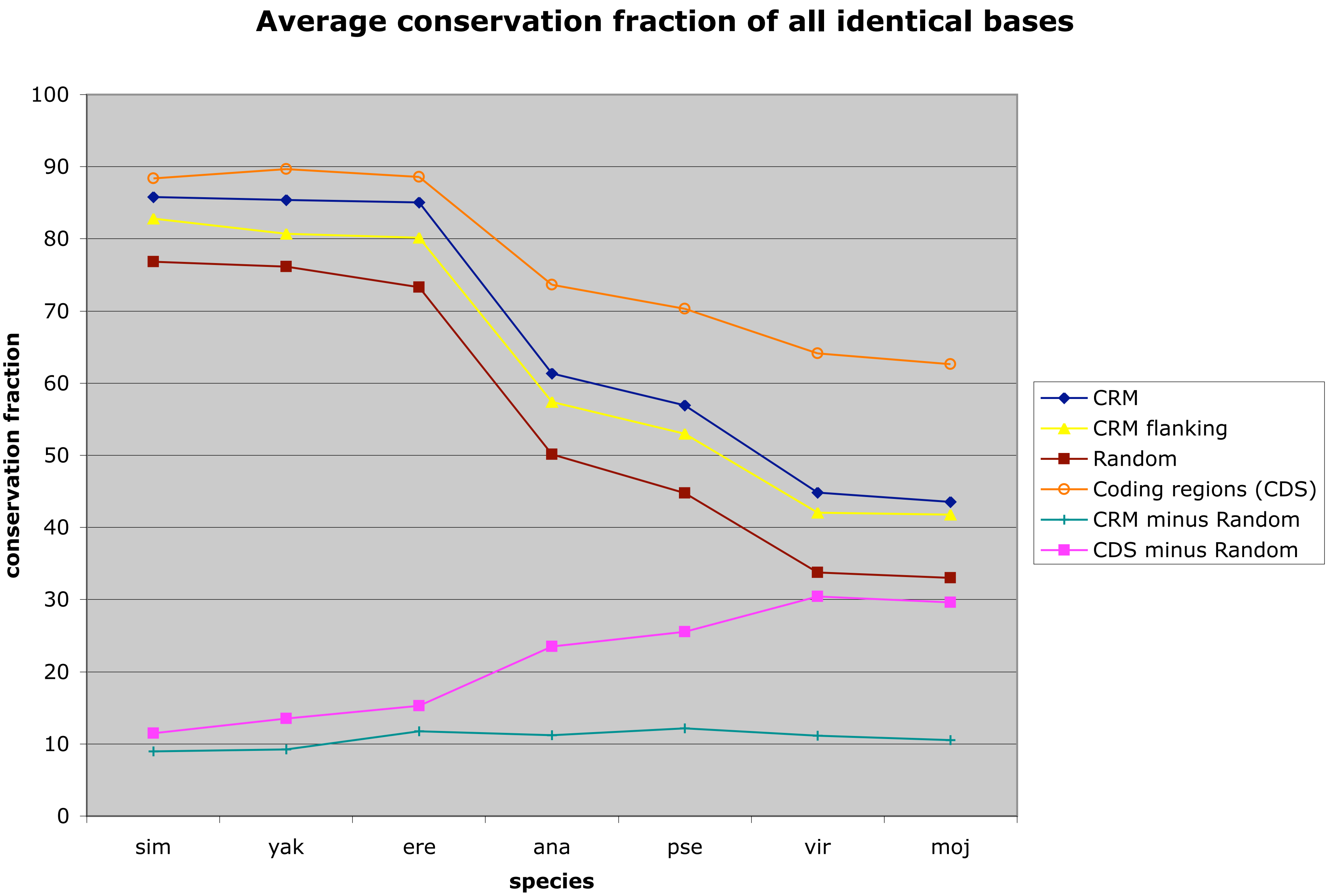

Figure S3-1: Average conservation fraction of all identical bases. See Fig. 3A and legend for details of each data type.

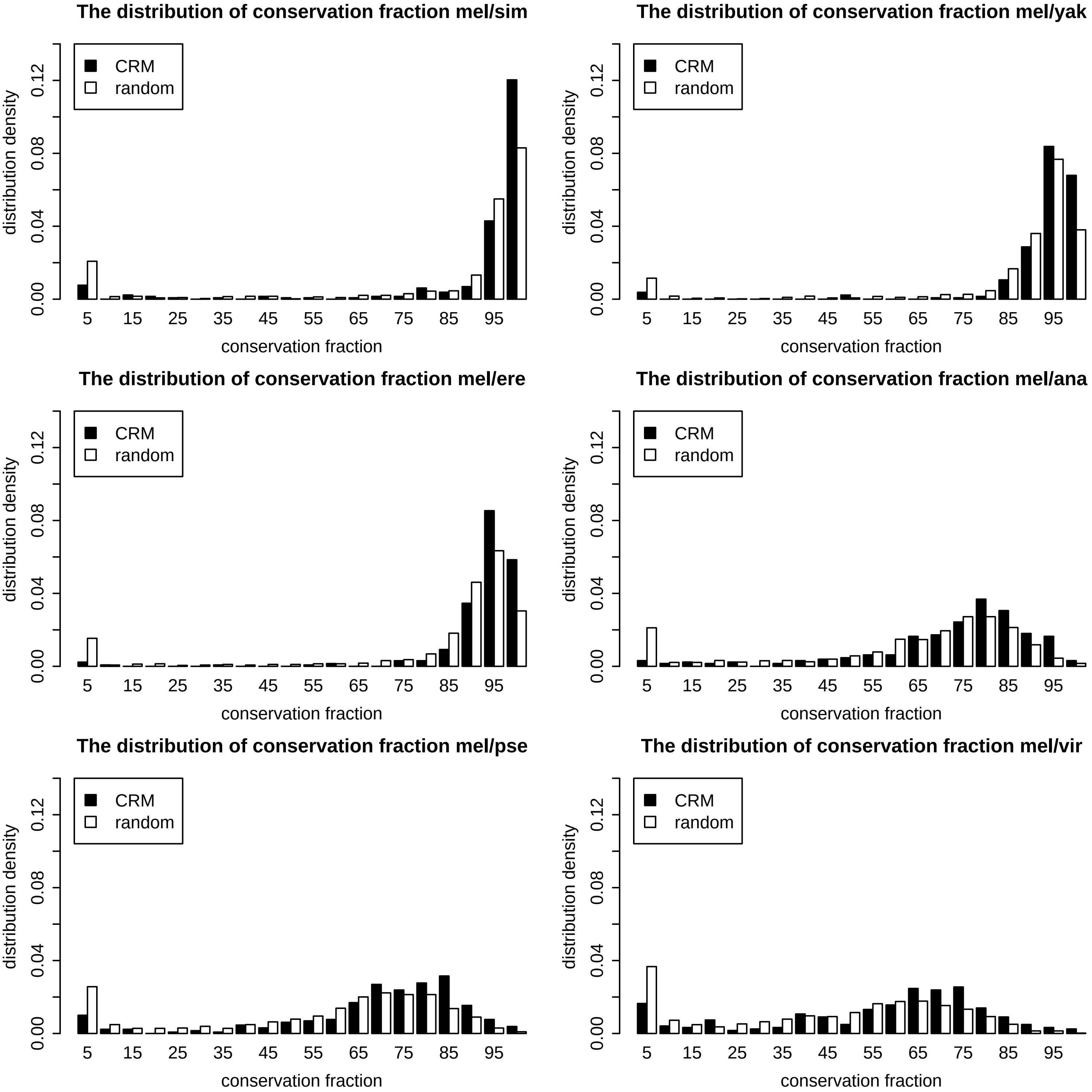

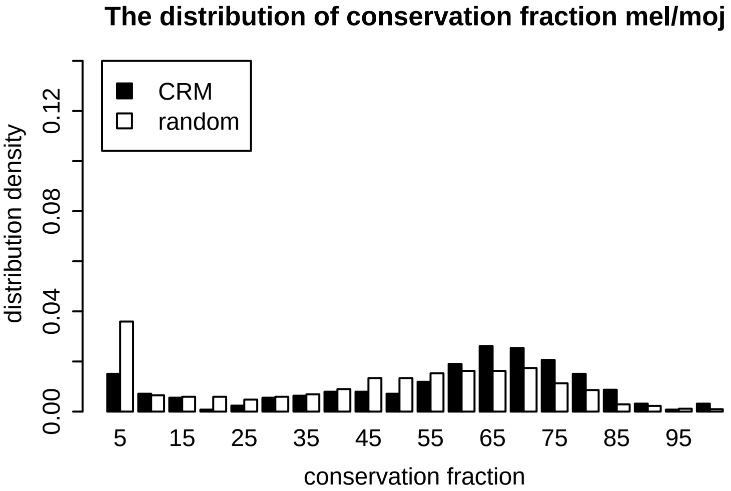

Figure S3-2. Histogram of the conservation fraction for CRMs (black bars) and random non−coding sequences (white bars) for D. melanogaster aligned with each of the other species.

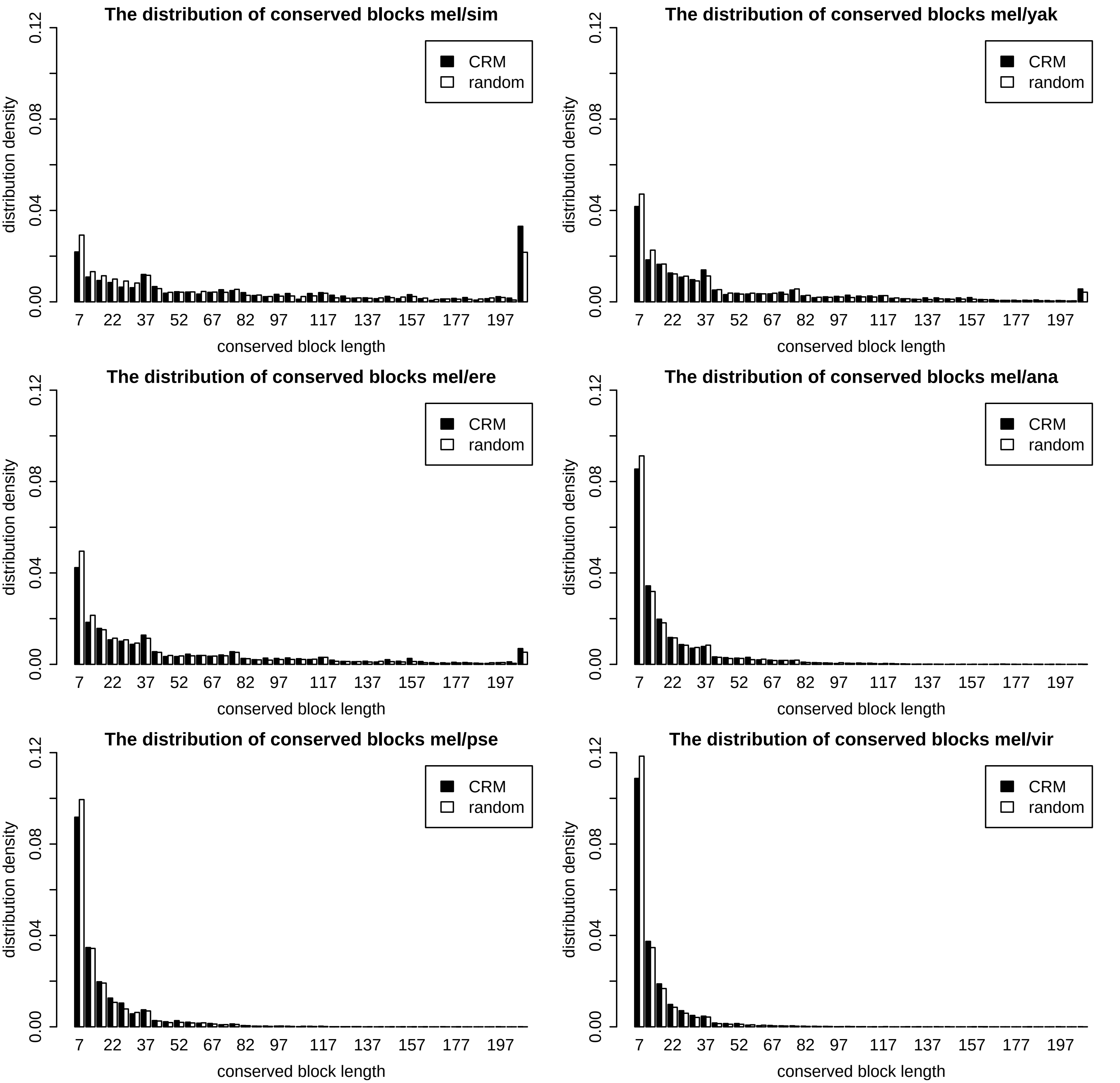

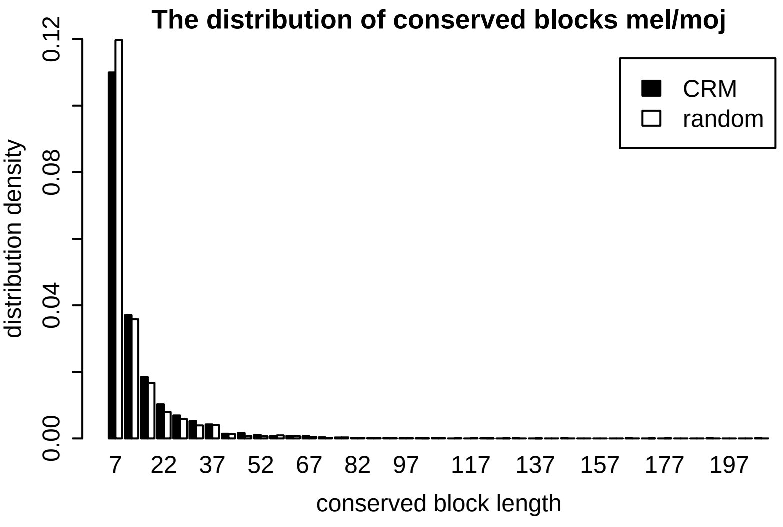

Figure S3-3. Histogram of the conserved block length for CRMs (black bars) and random non−coding sequences (white bars) for D. melanogaster aligned with each of the other species.

Li et al. Additional Data File 3

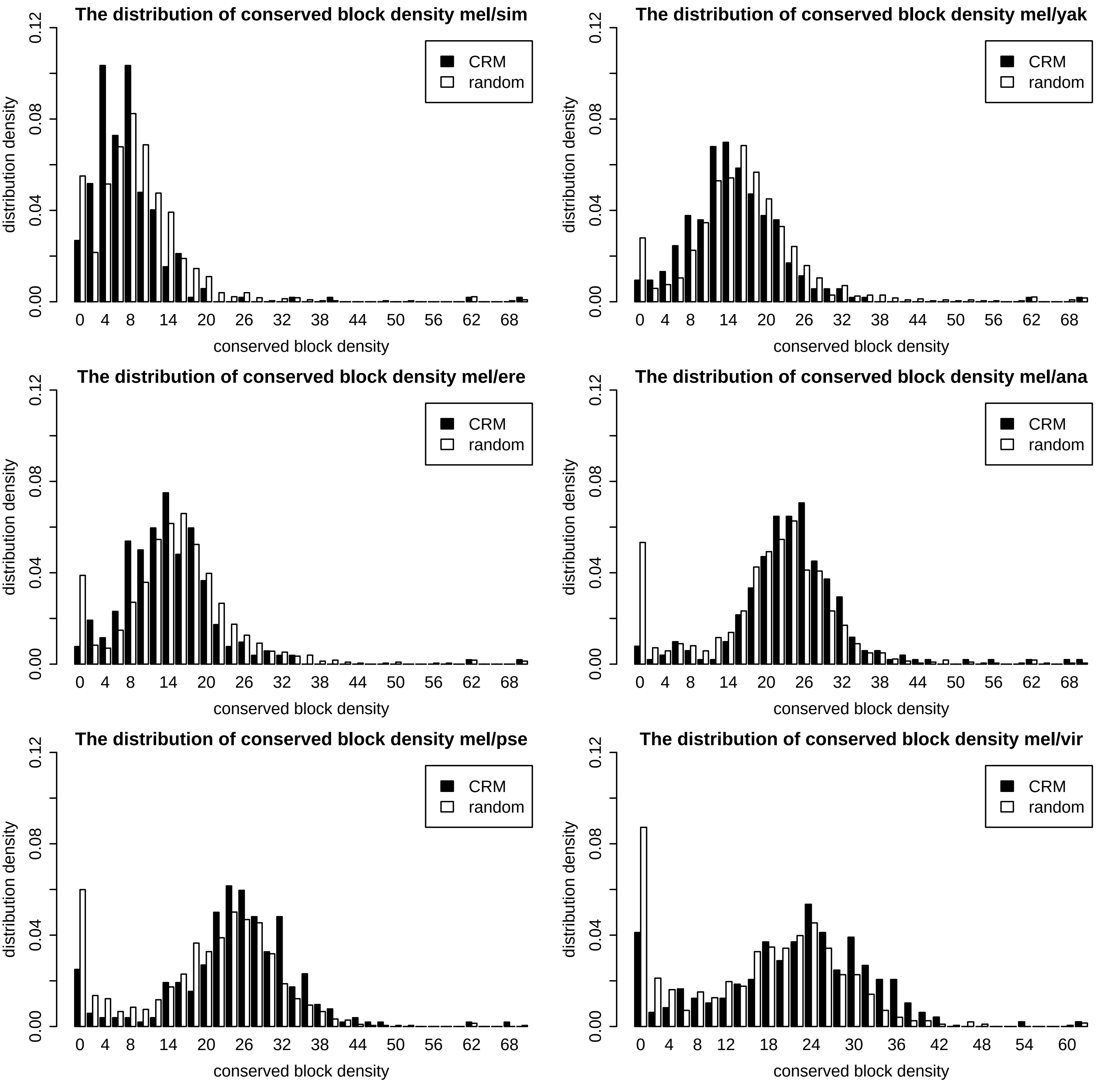

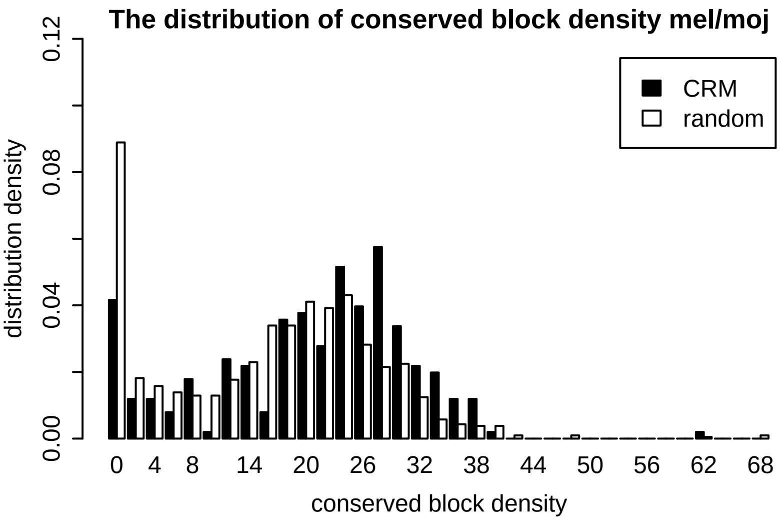

Figure S3-4. Histogram of the conserved block density for CRMs (black bars) and random non−coding sequences (white bars) for D. melanogaster aligned with each of the other species.

Li et al. Additional Data File 3
