# Supplementary material for: Large-scale analysis of transcriptional cis-regulatory modules reveals both common features and distinct subclasses
Source: Genome Biol. 2007 Jun 5;8(6):R101. doi: 10.1186/gb-2007-8-6-r101 (PMC2394749; doi:10.1186/gb-2007-8-6-r101)
Supplement: Additional data file 5 — Table S5-1 and Figure S5-1. [file gb-2007-8-6-r101-S5.pdf]

Additional Data File 5

In addition to the Manak et al. microarray data, we used the microarray data of Stolc et al. (2004) to examine whether or not the REDfly analysis CRMs are transcribed. Among the key differences between these two datasets are that (1) the Manak et al. experiments were done using total RNA while the Stolc et al. experiments used polyA-selected RNA and (2) the Manak et al. microarrays were more fully tiled, with over 15 times as many basepairs interrogated at 35 nucleotide resolution. As a result of the lower tiling density, only 149 of the CRMs have sequences represented on the Stolc et al. microarrays. Of these, 53% are transcribed (Table S5-1). We looked separately at CRMs that are promoter-proximal (within 500 bp of the TSS) and those that are more distant from the promoter.

Table S5-1: Transcribed CRMs from Stolc. et al.

**location**	**number of CRMs expressed**
promoter proximal/overlapping	6/19 (31.6%)
non-promoter	73/130 (56.2%)
total	79/149 (53.0%)
random sequence (non-promoter only)	3230/6400 (50.5%)

Surprisingly, the promoter-associated CRMs are significantly less likely to be transcribed (32% vs. 56%; Fisher's Exact $P < 0.04$). This is not seen in the Manak et al. data (data not shown); the difference is possibly due to the higher resolution/greater coverage of the probe tiling in that dataset. Because promoter proximity presented a possible confounding factor in the Stolc et al. dataset, for the remainder of the analysis we focused on only the promoter-distal CRMs.

To determine whether, like we saw for the Manak et al. microarrays, CRM transcription occurs at a significantly higher rate than background levels of transcription in noncoding sequences, we compared the percentage of transcribed CRMs with the percentage of transcribed size-matched random non-coding sequence fragments. Under our baseline assumption that the random non-coding sequence contains no CRMs (i.e., any observed transcription from these sequences is not from a CRM), we could not conclude with confidence that CRMs are transcribed at higher frequency than non-regulatory sequence (56% vs. 51%; Fig. S5-1, fraction=0%, $P < 0.10$ by two-sample test of proportions). However, as discussed in the text, assuming a 0% fraction of regulatory sequences in our randomly chosen set is probably incorrect. Therefore, we recalculated the significance of observing transcription of CRMs relative to background using different assumptions about the number of CRMs present in the randomly selected sequences

(see Fig. S5-1 legend). Figure S5-1 demonstrates that the association between being a CRM and being transcribed becomes significant ($P < 0.05$) at an estimated background fraction of 22% CRMs in non-coding sequence and highly significant ($P < 0.005$) at 51% background CRMs. Thus the Stolc et al. microaray data supports the conclusions from the Manak et al. microarrays, that CRMs are more frequently transcribed than random noncoding sequences.

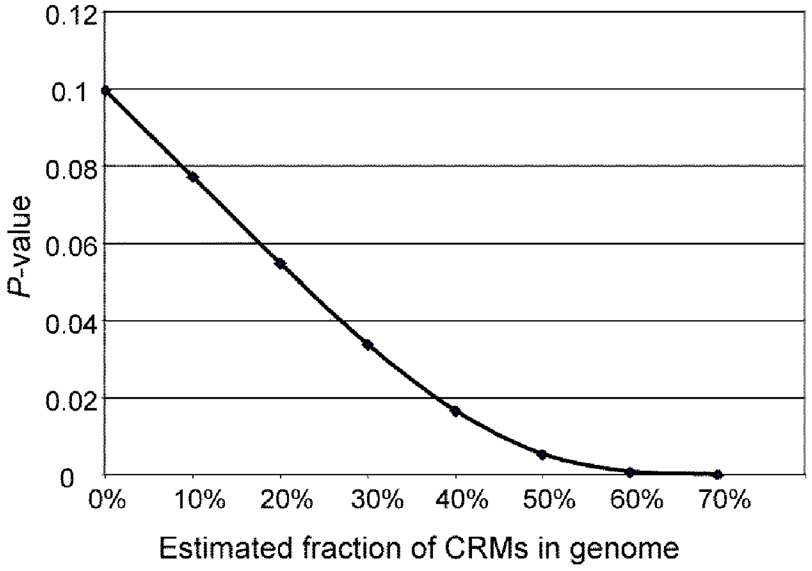

Figure S5-1. Statistical significance of increased association between being a CRM and being transcribed over being a transcribed non-coding non-regulatory sequence as a function of the fraction of CRMs contained within our randomly selected non-coding sequences. The *x*-axis gives estimated fractions of CRMs in the random sequences, *y*-axis gives *p*-values based on the two-sample *z*-test for proportions. **Methods:** Estimating the significance of CRMs being transcribed given different background frequencies of CRMs was performed as follows: (1) We reduced the total number of random sequences n_2 by the number we assumed to be real CRMs, *r*, to give a new total number $n_2' = n_2 - r$. (2) We assumed that the proportion of real CRMs that are transcribed (56%) would remain constant. Therefore, we reduced the "observed" number of transcribed random sequences *t* by 0.56*r* to give $t' = t - 0.56r$. (3) The two-sample test of proportions was then performed as in the text, using the original observed proportions of transcribed REDfly CRMs (n_1, p_1) and the modified values for the random sequences (n_2', $p_2' = t'/n_2'$).
