# Supplementary material for: Large-scale analysis of transcriptional cis-regulatory modules reveals both common features and distinct subclasses
Source: Genome Biol. 2007 Jun 5;8(6):R101. doi: 10.1186/gb-2007-8-6-r101 (PMC2394749; doi:10.1186/gb-2007-8-6-r101)
Supplement: Additional data file 6 — Differences between the original FTT and the revised FTT-Z (Figures S6-1 and S6-2) and CRM subsets with significant FTT-Z scores (Table S6-1). [file gb-2007-8-6-r101-S6.pdf]

Table S6-1:

**Tissue/Stage**[a]	**adjusted *P*-value**[b]
blastoderm	**0.00090**
ectoderm	**0.00472**
endoderm	0.13541
eye	1
mesoderm	0.79046
neuronal	1
reproductive system	1
wing	1
adult	**0.00714**
embryo	**0.00002**
larva	1

[a]See Zhu et al. Only subsets with ≥15 CRMs are included.
[b]rank sum test, Bonferroni-corrected *P*-values. **Bold** type indicates significance.

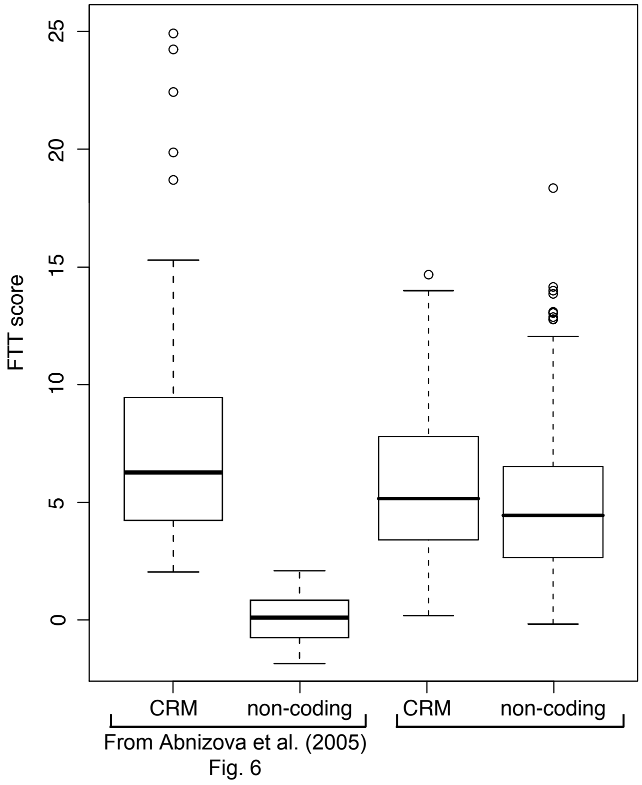
FTT score
0
5
10
15
20
25
CRM
non-coding
CRM
non-coding
From Abnizova et al. (2005)
Fig. 6

Fig S6-2

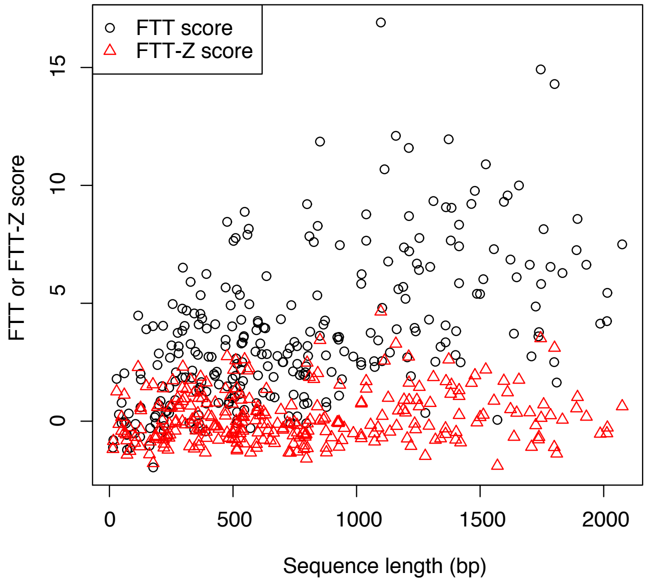
FTT score
FTT-Z score
FTT or FTT-Z score
0
5
10
15
0
500
1000
1500
2000
Sequence length (bp)

Figure S6-1: Results using the original Fluffy-Tail Test of Abnizova et al. (2005). Boxplots indicate the median (heavy bar) and first and third quartiles of the data (boxed area). Columns one and two show data reproduced from Figure 6 of Abnizova et al. (2005), which shows FTT scores for 60 *Drosophila* CRMs and an equal number of non-coding, non-regulatory sequences. We were concerned that the poor separation we observed between CRM and non-regulatory sequences using the FTT-Z (Fig. 5, columns 1 and 2) might be due to our modifications to the FTT. Therefore, we conducted additional tests using the original version and the same 60 CRMs used by Abnizova et al. (2005). Surprisingly, we again found the separation between the CRMs and random non-coding sequence to be poor but significantly different (columns 3 and 4; rank sum test $P <$ 5.24E-06). We attribute the difference between our results and those reported by Abnizova et al. (2005) to the fact that we used much greater numbers of random sequences (see Methods).

Figure S6-2: The FTT score is confounded with sequence length. FTT scores plotted versus the length of the sequence scored show that the score increases with increasing sequence length (black circles, $r = 0.57$). A similar plot using the FTT-Z score shows that this correlation is dramatically weakened with the revised method (red triangles; $r = 0.14$).
